# Talk to Me, Not Just My Parent: Teen and Caregiver Perspectives on Implementing Screening, Brief Intervention, and Referral to Treatment Equitably in Pediatric Inpatient Settings for Teens with Chronic Illness

**DOI:** 10.3390/children13091184

**Published:** 2026-09-02

**Authors:** Faith Summersett Williams, Sarah Welch, Ella Kuffour, Emily Lynott, Sheridan Grettenberger, Kennedy Curtis, Yiyang Liu, Ruth Debono, Maria H. Rahmandar, Sara Becker

**Affiliations:** 1 Division of Adolescent and Young Adult Medicine, Ann & Robert H. Lurie Children’s Hospital of Chicago, Chicago, IL 60611, USA; ellakuffour2025@u.northwestern.edu (E.K.); elynott@luriechildrens.org (E.L.); sgrettenberger@luriechildrens.org (S.G.); kecurtis@luriechildrens.org (K.C.); yiyliu@luriechildrens.org (Y.L.); rdebono@luriechildrens.org (R.D.); mrahmandar@luriechildrens.org (M.H.R.); 2 Department of Pediatrics, Feinberg School of Medicine, Northwestern University, Chicago, IL 60208, USA; 3 Department of Emergency Medicine, Feinberg School of Medicine, Northwestern University, Chicago, IL 60208, USA; sarah.welch@northwestern.edu; 4 Department of Psychiatry, Feinberg School of Medicine, The Center for Dissemination and Implementation Science (CDIS), Northwestern University, Chicago, IL 60208, USA; sara.becker@northwestern.edu

**Keywords:** adolescents with chronic medical conditions, caregivers, alcohol and other drug use, SBIRT, inpatient hospitalization, pediatric hospitals

## Abstract

**Highlights:**

**What are the main findings?**

- Adolescents with chronic medical conditions (A-CMCs) and caregivers broadly supported inpatient SBIRT as important and appropriate, but its acceptability hinged on how it was delivered.
- Sociopolitical context such as stigma around “having a problem” versus normalized casual alcohol and drug (AOD) use, blurred lines between prescribed and nonmedical substances. Structural conditions such as limited behavioral health referral sources also shaped A-CMCs and caregivers’ willingness to disclose AOD use and their ability to benefit from SBIRT’s brief intervention and referral components.

**What are the implications of the main findings?**

- Inpatient SBIRT for A-CMCs should adopt developmentally appropriate explanations of confidentiality and its limits, patient-centered workflows, and flexible timing/modality of SBIRT to build the relational safety needed for honest AOD use disclosure.
- Because referral alone may be insufficient given fragmented care and limited behavioral health access, inpatient hospitals should pair SBIRT screening with integrated, coordinated follow-up delivered in parallel with chronic illness care. Future implementation and co-design research should explicitly evaluate whether these adaptations advance equity or inadvertently widen gaps across racial, ethnic, disability, and socioeconomic groups.

**Abstract:**

**Background/Objectives:** While screening, brief intervention, and referral to treatment (SBIRT) is a widely recommended evidence-based approach for early detection and intervention for alcohol and other drug (AOD) use, limited guidance exists for implementing SBIRT among hospitalized adolescents with chronic medical conditions (A-CMCs). This exploratory qualitative study examined A-CMC and caregiver perspectives on factors that may shape the acceptability, feasibility, and equitable implementation of a proposed inpatient SBIRT approach for A-CMCs. **Methods:** Two separate focus groups were conducted in an urban pediatric hospital in 2023 with A-CMCs aged 13–18 (*n* = 7), who had a history of hospitalization for their medical condition, and their caregivers (*n* = 6). Data were coded using thematic analysis guided by the Consolidated Framework for Implementation Research (CFIR) and the Health Equity Implementation Framework (HEIF), which captured implementation and equity-relevant determinants, respectively. **Results:** Although A-CMCs and caregivers recognized the importance of SBIRT within hospital settings, its acceptability hinged on the conditions of its delivery. The timing, relevance to current health needs, and modality of screening shaped an A-CMC’s willingness to disclose AOD use. Clinician communication style, including the use of a nonjudgmental tone and clear parameters for confidentiality, were also indicated as crucial for SBIRT delivery. Broadly, participants noted the significant impact that the sociopolitical context (e.g., stigma) and structural factors (e.g., financial burden) had on a family’s ability to benefit from SBIRT. **Conclusions:** In this exploratory qualitative study, participants identified confidentiality-forward, patient-centered workflows, and accessible follow-up supports as potentially important considerations for inpatient SBIRT among A-CMCs. These findings generate hypotheses for future co-design and implementation research across diverse pediatric inpatient settings.

## 1. Introduction

Alcohol and other drug (AOD) use in adolescence remains a major public health concern, associated with increased risk for psychiatric comorbidity, altered neurodevelopment and cognition, poorer educational attainment, and preventable injury and mortality [1,2,3,4,5]. Despite these risks, adolescents with chronic medical conditions (A-CMCs) engage in AOD use at equal or greater rates than their healthy peers [6,7], are more likely to initiate AOD use prior to 14 years of age and show increased risk for developing a substance use disorder across adolescence and emerging adulthood [7]. The form and level of AOD use among A-CMCs varies relative to the complexity, severity, and type of chronic medical condition [7,8]. However, it is unclear how the frequency and degree of engagement with inpatient care necessitated by those conditions contributes to this range in AOD use by A-CMCs, as well as the reasons and motivations behind their AOD use [6,7,8]. While some A-CMCs are driven by developmentally typical desires for autonomy and experimentation, others describe using substances to facilitate peer integration or to manage pain, stress, and disease-related symptoms [9,10,11].

AOD use poses unique and amplified risks for A-CMCs. AOD use may interact with prescribed medications, compromise laboratory interpretation, worsen disease control, and undermine medication adherence [8,12,13]. For A-CMCs managing complex treatment regimens, AOD use can complicate self-management and transition readiness, particularly during late adolescence when healthcare transfer and increasing autonomy coincide with heightened risk-taking behaviors [7,14]. Importantly, many A-CMCs experience fragmented healthcare contact during this developmental window, including irregular primary care attendance, frequent inpatient hospitalizations related to their medical condition, and disrupted continuity as they transition to adult systems [15,16].

Screening, brief intervention, and referral to treatment (SBIRT) is an evidence-based approach designed to identify and address AOD use within healthcare settings [17,18]. SBIRT is recommended for routine adolescent care, with primary care and emergency department settings being common SBIRT targets [18]. However, implementation in these settings remains inconsistent, and validated screening tools and follow-through procedures are not universally used [19]. Moreover, A-CMCs may engage with primary care irregularly during transitional periods [15], and high patient acuity and volume in emergency departments can limit systematic implementation [20]. Consequently, the implementation of SBIRT in ambulatory and primary care settings alone may limit the degree to which A-CMCs receive routine AOD use screening and their ability to benefit from SBIRT in such encounters.

Pediatric inpatient hospitalization represents a potentially high-impact touchpoint for AOD use screening for A-CMCs. A-CMCs comprise the bulk of pediatric inpatient hospital stays in the United States, and hospitalization offers direct access to A-CMCs at moments when medication management, disease exacerbation, and care planning are actively occurring [9,21]. Yet little focus has been directed towards understanding the current landscape of SBIRT implementation within pediatric inpatient units, much less for A-CMCs receiving care in those settings. Additionally, hospitalization is also an emotionally charged and relationally complex context. A-CMCs are typically physically unwell, overwhelmed by admission procedures, and accompanied by caregivers [11,20]. Conversations about AOD use in this setting therefore intersect with issues of confidentiality, autonomy, stigma, family dynamics, institutional trust, and the need to triage competing clinical and psychosocial concerns during hospitalization [20]. While these complexities have long been identified as barriers to the use of SBIRT by clinicians within primary care settings, the distinct ways in which they present and may be navigated in the pediatric inpatient context require further study [22,23,24]. Emerging evidence suggests that implementation challenges in acute care settings are not solely logistical but also relational and structural [20,25]. For A-CMCs in particular, AOD screening is embedded within broader experiences of chronic illness management, disability accommodation, healthcare system navigation, and sociopolitical influences shaping stigma and disclosure [9,26]. A-CMC willingness to engage honestly in screening may depend on perceptions of confidentiality, clinician communication style, prior experiences of being heard or dismissed, and the degree to which screening is meaningfully integrated into their care rather than presented as a checklist exercise [9,27,28].

Beyond increasing access to SBIRT, equitable implementation seeks to ensure that evidence-based interventions are delivered in ways that are responsive to the needs, preferences, and lived experiences of populations that have historically experienced disparities in healthcare [29]. Equitable implementation refers to delivering evidence-based interventions in ways that actively reduce, rather than reproduce, inequities in access, quality, and outcomes among groups who experience structural disadvantage in the healthcare system [29,30]. In the context of SBIRT, equitable implementation means ensuring that screening and brief interventions for AOD use are systematically offered and meaningfully beneficial to A-CMCs and their families, not only to those who already receive high-quality, well-resourced care. This includes attention to how SBIRT addresses stigma, time pressures, communication needs, and prior experiences of discrimination or dismissal that can shape whether A-CMCs and caregivers perceive SBIRT as fair, respectful, and trustworthy. Without attention to these contextual factors, implementation strategies intended to improve uptake may inadvertently reinforce existing inequities in care [29].

Guided by this concept of equitable implementation, the present qualitative study was designed to understand how SBIRT could be integrated into care for A-CMCs in ways that promote equity in detection and response to AOD use. By centering caregiver and A-CMC perspectives, this study sought to answer the following research questions: (1) What do A-CMCs and caregivers identify as facilitators and barriers to inpatient SBIRT disclosure and engagement? (2) How do confidentiality, timing, and relational dynamics shape perceived acceptability? Because caregiver presence can inhibit adolescent candor about sensitive topics, we intentionally recruited A-CMCs and caregivers as independent, non-dyadic samples. This design was intended to elicit complementary rather than paired accounts, allowing each group to speak freely without needing to reconcile their perspectives with a specific family member’s. Thus, this study sought to identify equity-relevant barriers and facilitators, such as concerns about being singled out because of a medical condition, fears of judgment, or preferences for how clinicians discuss AOD use, that could inform future SBIRT implementation research in pediatric specialty settings. We explored preferences regarding modality, timing, confidentiality, relational approach, and follow-up pathways to identify contextually relevant and equitable implementation determinants that will inform future studies focusing on the delivery of SBIRT in pediatric inpatient settings. Throughout this study, we distinguish among four related but distinct constructs: acceptability (participants’ appraisal of whether inpatient SBIRT is appropriate, tolerable, and respectful of their needs); perceived feasibility (the practicality of delivering SBIRT given inpatient workflow, timing, and resource constraints); equity-relevant determinants (equity-derived contextual factors, such as stigma, structural barriers, disability, and sociopolitical context, that may shape whether SBIRT is experienced equitably); and proposed adaptations (specific modifications to screening, delivery, or follow-up that participants suggested in response to these determinants). We grounded this study in the Consolidated Framework for Implementation Research (CFIR) and the Health Equity Implementation Framework (HEIF). The CFIR is a widely used conceptual framework to theorize determinants of implementation [31]. It provides a comprehensive structure for assessing implementation determinants across domains including intervention characteristics, inner and outer setting, characteristics of individuals, and implementation processes [32]. While the CFIR was developed as a comprehensive structure to evaluate barriers, facilitators, and constraints to implementation in diverse settings, it and other determinant frameworks have been critiqued for insufficient attention to structural and sociopolitical determinants of implementation [29,30]. The HEIF extends determinant frameworks by explicitly incorporating patient culturally relevant factors, clinical encounter dynamics, physical structures, and broader sociopolitical contexts that shape equitable implementation [33,34]. Together, the CFIR and HEIF offer a lens for understanding how confidentiality practices, stigma, structural barriers, disability inclusion, and institutional culture influence patient preferences for and perceptions of the acceptability of inpatient SBIRT for A-CMCs. By combining the CFIR and HEIF, this exploratory qualitative study elicited A-CMC and caregiver perspectives on multi-level factors that may shape the acceptability, feasibility, and equitable implementation of inpatient SBIRT for A-CMCs. These participant-identified determinants are intended to generate hypotheses and potential considerations for future co-design and testing.

## 2. Materials and Methods

Design and Setting:

This qualitative study was based at Ann & Robert H. Lurie Children’s Hospital of Chicago, a nationally ranked, freestanding pediatric academic medical center in Chicago, Illinois, and the primary pediatric teaching affiliate of Northwestern University Feinberg School of Medicine. During the study period, the hospital did not conduct universal SBIRT screening for A-CMCs; however, there was established infrastructure to address AOD use concerns identified through usual care, including social work consultation services and an outpatient substance use treatment program that served as a referral pathway for adolescents requiring specialty care. We recruited A-CMCs who were hospitalized on the Hematology, Oncology, Neuro-Oncology, and Stem Cell Transplantation unit, as well as the Nephrology, Cardiology, and Gastroenterology units. Separate virtual focus groups were conducted with A-CMCs aged 13–18 and parents or caregivers of A-CMCs in 2023. The hospital’s Institutional Review Board approved all procedures (IRB 2022-5048). This qualitative study’s design, conduct, and reporting were informed by relevant domains of the Standards for Reporting Qualitative Research (SRQR), including research team characteristics and reflexivity, context, sampling strategy, data collection procedures, analytic approach, and trustworthiness, as detailed in the sections below [35].

Participants:

To participate in the study, A-CMCs had to be aged 13–18 years, speak English, and have an inpatient hospitalization for a chronic medical condition (e.g., sickle cell disease, diabetes, asthma) within the last five years. Caregivers had to be English-speaking parents or legal guardians of an A-CMC with a hospitalization in the last five years. Caregivers and A-CMCs were recruited as independent samples and were not matched dyads. We used convenience sampling to recruit A-CMCs and caregivers as independent, non-dyadic samples. Recruitment materials were distributed electronically and in relevant clinical settings. The adolescent flyer included a QR code that directed interested adolescents to a brief, password-protected Google Forms study-interest survey, which collected contact information. The caregiver flyer provided a telephone number for a study research assistant; interested caregivers contacted the research assistant directly to learn more about the study. Caregiver recruitment relied exclusively on this telephone-based pathway. No online contact form was used to recruit caregivers. For both groups, a member of the research team described the study, confirmed eligibility, answered questions, and assessed availability for a virtual focus group.

Seventy-six adolescents submitted the QR-code-linked study interest survey, and six caregivers contacted the study research assistant. Because recruitment was voluntary and flyer-based, the number of individuals who viewed recruitment materials or were otherwise exposed to the study invitation could not be determined. Seven adolescents participated in the adolescent focus group, and all six caregivers who contacted the study research assistant participated in the caregiver focus group. We did not systematically record eligibility outcomes, formal refusals, or reasons for nonparticipation among adolescents who submitted the interest survey but did not participate; therefore, nonparticipation should not be interpreted as refusal. Nonetheless, the substantial gap between the 76 adolescents who expressed interest and the 7 who ultimately participated raises the possibility of selection bias, such that participating adolescents may have differed systematically from non-participants in comfort discussing sensitive topics, scheduling flexibility, caregiver support for participation, or trust in the research team and health system; these possible differences should be considered when interpreting the transferability of adolescent-specific findings. Participants received a $25 electronic gift card in acknowledgment of their time and expertise.

We did not set a prespecified sample-size target or use a formal thematic saturation stopping rule. Instead, sample adequacy was considered in relation to information power, the focused study aim, the specificity of the participant population, the use of theoretically informed focus group guides, and the depth of discussion generated within each focus group [36]. The resulting dataset was considered suitable for an exploratory, hypothesis-generating analysis of A-CMCs and caregiver perspectives on inpatient SBIRT. Because only one focus group was conducted per participant category, we were unable to confirm thematic sufficiency, that is, whether an additional focus group within each category would have surfaced substantially different or divergent themes. This constraint should be considered when interpreting the completeness of the themes presented below.

Data Collection:

The core research team included scientists and clinicians with expertise in child and adolescent psychology, dissemination and implementation science, qualitative methods, and addiction medicine, including individuals who identify as White, Black, and Latina, and Asian. These identities confer both institutional privilege and lived experiences of navigating healthcare systems as members of differently racialized and gendered groups, which informed the team’s commitments to equity but also created potential blind spots. Most of the team were affiliated with the same pediatric hospital system as participants’ clinical care but were not directly responsible for the participants’ medical treatment. The principal investigator, a female pediatric clinical psychologist with experience caring for A-CMCs and conducting implementation science research, facilitated both focus groups. Prior to data collection, the team engaged in reflexive discussion of how their social locations and professional roles might shape data collection and interpretation, particularly given the study’s focus on adolescent AOD use, SBIRT, health equity, and chronic illness. The team also engaged in reflexive consideration of concerns about under-recognition of AOD use in medical settings and commitments to health equity and revisited these reflections during analysis to guard against over-emphasizing anticipated themes while remaining open to disconfirming evidence. Throughout data collection and analysis, the research team acknowledged that their clinical and research experiences could shape interpretation of participants’ perspectives. To promote reflexivity, coding decisions were discussed during regular analytic meetings, divergent interpretations were explored through consensus discussions, and themes were refined collaboratively to ensure they remained grounded in participants’ narratives rather than researchers’ assumptions.

Because the PI was affiliated with the same pediatric institution where many of the A-CMCs received care, the team took deliberate steps to minimize perceived coercion and hierarchical pressure. Families were informed that the PI was acting in a research (not clinical) role for this study, that participation was entirely voluntary, and that choosing not to participate (or sharing critical views of SBIRT or the healthcare system) would not affect their medical care. At the beginning of each group, the PI emphasized confidentiality, invited a range of perspectives, and explicitly stated that there were no right or wrong answers, to counteract assumptions that the facilitator held the correct view. During the focus group discussion, the PI used specific strategies to redistribute voice and reduce power asymmetries. The PI asked open-ended, non-leading questions (using the focus group guide), validated diverse experiences, acknowledged when topics might feel sensitive; and intentionally invited contributions from quieter participants while avoiding over-engagement with more vocal participants. Separate groups were conducted for caregivers and A-CMCs to lessen intra-family power dynamics and allow A-CMCs, in particular, to speak freely about their preferences and concerns related to SBIRT in the context of chronic illness.

Focus group guides were grounded in the CFIR and HEIF to elicit multi-level determinants of inpatient SBIRT implementation for A-CMCs. Guided by the CFIR, we elicited feedback related to Intervention Characteristics (e.g., perceived appropriateness of SBIRT during hospitalization, complexity of screening workflows, and preferences for delivery modality), Characteristics of Individuals (e.g., knowledge of SBIRT, beliefs about AOD use and disclosure, and perceived consequences of disclosure), and the Implementation Process (e.g., preferred timing and personnel for screening, strategies to support engagement, and recommendations for follow-up/referral), while also noting contextual influences (e.g., aspects of the inner and outer setting) when they emerged. Guided by the HEIF, we elicited feedback on cultural and contextual determinants of equitable implementation, including the clinical encounter and relational safety (e.g., respect, communication style, and trust), patient culturally relevant factors (e.g., confidentiality expectations and family dynamics), sociopolitical influences (e.g., stigma and social norms), and physical structures shaping access and follow-up (e.g., transportation, parking, and availability of behavioral health resources).

Because universal SBIRT was not implemented for A-CMCs at the hospital during the study period, the focus groups were designed to elicit anticipatory perspectives on potential inpatient SBIRT implementation rather than to evaluate experiences with an existing routine SBIRT program. Before implementation-focused questions, the facilitator briefly described the core components of SBIRT and invited participants to consider how these practices might be introduced and delivered during hospitalization. Participants were discouraged from sharing personal AOD use experiences; instead, discussion focused on anticipated acceptability, concerns, barriers, facilitators, and preferences related to a proposed routine inpatient SBIRT approach.

Focus group sessions were virtual 60 min zoom sessions and both sessions were audio recorded and visually documented using live graphic facilitation (Appendix A.3 and Appendix A.4). The live graphic recording was displayed to participants at the end of the session, and the facilitator invited participants to confirm, clarify, or add to the themes visually captured in real time. This served as a real-time validation check of the session’s visual summary, allowing participants to confirm that the graphic recording accurately reflected what was discussed. This process is distinct from member-checking of final analytic themes because coding and theme development occurred after data collection and the final themes presented in this manuscript were not returned to participants for review. Audio recordings were transcribed and de-identified by a professional transcription service. De-identified transcripts were received by the study team and uploaded to Dedoose for analysis [37].

Analysis:

Two trained qualitative coders conducted thematic analysis of session transcripts using a combination of deductive (framework-informed) and inductive coding. The lead analyst conducted two trainings with the coders prior to beginning coding. These trainings focused on thematic qualitative analysis, applying those methods to this study, and training on the codebook, qualitative software, and the process for code review and reconciliation. After the trainings, coders were given time to practice with the codebook and the lead analyst provided iterative feedback until codes were being applied appropriately and consistently. Coders held research positions within the study team and neither they nor the lead analyst had any direct clinical or supervisory relationship with participants, minimizing potential power differentials between coders and participants. Throughout the coding process, both coders maintained reflexive memos documenting their reactions, assumptions, and interpretive decisions, which were reviewed regularly with the lead analyst through weekly meetings and code reconciliation tracking sheets that included space for coder notes and thoughts.

The lead analyst, an evaluator with expertise in qualitative methods, oversaw the coding process and led the transition from initial codes to final themes. This involved reviewing both coders’ independent coding for convergence and divergence, facilitating consensus discussions to resolve discrepancies, and synthesizing overlapping or related codes into higher-order themes. The lead analyst’s background in public health balanced the experience of those on the team with clinical experience. The themes were presented to the rest of the project team for further discussion and refinement. This iterative, team-based approach to theme development was intended to enhance analytic rigor and reduce the influence of any single coder’s or analyst’s positionality on the final results.

To guard against the deductive frameworks overdetermining the coding process, coders were trained to view the codebook as an initial guide, but not an exhaustive list of concepts present in the data. Coders were encouraged to keep track of excerpts that did not “fit” the constructs in the CFIR and HEIF. These new constructs were reviewed and discussed by the coding team and, if deemed to rise to the level of a relevant code, added to the codebook. Both focus group transcripts were blind double-coded. In lieu of quantitative inter-rater reliability statistics, this study used a consensus approach. Code application was reviewed by the two coders for concordance and any disagreement on placement or code was discussed between the two coders with additional guidance provided by review of the codebook definitions and the lead qualitative analyst, as needed (Appendix A.1). All codes and placements were finalized according to consensus reached through this process. All excerpts with a given code applied were summarized and categorized into initial themes. This process was repeated for all codes. These initial themes were then discussed by the entire project team to identify overarching themes and map them to CFIR and HEIF constructs to identify key implementation determinants (Appendix A.2).

Demographic Information:

Thirteen individuals participated across two focus groups: seven adolescents and six caregivers. Four participants reported a racial identity of White, one identified as Black or African American, two identified as “Something Else,” and three identified their ethnicity as Hispanic or Latino. Adolescent gender identities included female (5 participants), trans-male (1 participant), and non-binary/gender expansive (1 participant). Mean adolescent age was 14.6 years, with three enrolled in middle school and four enrolled in high school. Adolescents reported receiving care at the hospital for cancer, kidney transplants, gastrointestinal health, and other chronic health conditions. Only 1 out of the 7 adolescents confirmed that there was a time in the past 12 months when they needed to see a doctor but could not because of the cost.

Among caregivers, four identified as White, two identified as Black or African American, and one identified as Hispanic or Latino. Most caregivers identified as female (5 participants). Caregiver ages ranged from 30–39 (1 participant) to 40–49 (3 participants) and ≥50 (2 participants). Caregivers reported their A-CMC was diagnosed with epilepsy, liver disease, congenital heart condition, sickle cell, asthma, hypothyroidism, hyperinsulinism and ulcerative colitis. No caregiver reported there was a time in the past 12 months their child needed to see a doctor but could not because of the cost. Participant characteristics are summarized in Table 1.

Themes are presented in an order that moves from broader sociopolitical and culturally relevant influences on AOD use and disclosure (Theme 1) to structural and workflow conditions shaping the acceptability of inpatient SBIRT for A-CMCs (Theme 2), to the relational dynamics of confidentiality, autonomy, and trust within SBIRT clinical encounters (Theme 3), and finally to the structural barriers and enablers that affect follow-up after substance use concerns are identified (Theme 4). This organization reflects the multilevel perspective of the HEIF and CFIR, spanning societal context, patient and deliverer needs and resources, intervention characteristics, and the clinical encounter itself [32,33]. Unless otherwise noted, findings specific to SBIRT reflect participants’ hypothetical or anticipatory perspectives on a proposed inpatient implementation approach, rather than direct experiences receiving SBIRT or personal experiences of AOD use. Furthermore, findings concerning trust, communication, confidentiality, and prior healthcare encounters reflect participants’ lived experiences in healthcare settings.

Theme 1: Sociocultural and Chronic Illness Context Shaping AOD Disclosure. This theme reflects the HEIF’s Societal Context and Culturally Relevant Factors domains and CFIR constructs in the Outer Setting domain related to community awareness and beliefs about AOD use, highlighting how stigma, normalization, social media, family modeling, and chronic illness context shaped how A-CMCs and caregivers understood AOD use and whether it felt safe or necessary to disclose.

Adolescents and caregivers described AOD use disclosure as shaped by broader societal forces that influence norms, perceived acceptability, and willingness to seek help. These forces included stigma, social media exposure, and the normalization of substance use in adolescents’ environments. Caregivers highlighted a perceived double standard in peer culture: casual AOD use was often normalized, whereas adolescents with problematic use faced strong stigma. One caregiver commented, “*There’s certainly a lot of glorification around the use. But a lot of stigma around actually having a problem with it…I know that teens are very quick to engage with their friends about casual usage…but when it comes to, “Do I have a problem with it?” Then yes, there’s a stigma attached to that. And also, getting treatment for it*” *(Caregiver 3)*. Caregivers also emphasized how the broader environment made substances “*easily accessible*” and normalized, including the perception that “*it’s no big deal to pop a pill*” or use other substances. Caregivers also pointed to the prevalence and influence of social media as a powerful force shaping what A-CMCs are exposed to, what they believe is common, and what is portrayed as desirable or acceptable. For instance, one caregiver reflected, “*the prevalence of social media is a whole other aspect, of what they see or what they might think is cool*” *(Caregiver 1)*, while another noted “*definitely social media plays a big part in it, and what’s cool and what’s not cool, and all of that*” *(Caregiver 6)* and another described “*It’s like it’s part of growing up. It’s like you see it in the movies, and it’s an experience. But it’s one that can be dangerous for my [child]*” *(Caregiver 4)*. These societal forces functioned primarily as barriers to SBIRT’s implementation, by shaping whether A-CMCs view disclosure as safe and whether they interpret screening as supportive versus punitive. In particular, participants suggested that stigma around ‘having a problem’ may suppress honest reporting and willingness to accept referral, underscoring the need for nonjudgmental SBIRT delivery and normalization of help-seeking. These narratives show how broader cultural messages and structural stigma may shape whether A-CMCs view disclosure in SBIRT as safe, shameful, supportive, or punitive.

Within this broader context, A-CMCs and caregivers also described how family environments and chronic illness care shape perceptions of AOD use. A-CMCs highlighted the role of family environment and parental behavior in shaping their exposure to and perceptions of AOD. One A-CMC reflected, “*I think a lot of kids in our generation at least have grown up with parents who… drink more than usual. It’s more of a kind of a more normal-ish thing than it used to be*” *(Teen 2)*. A-CMCs perceived that many youth were growing up in contexts where adult drinking is more visible, and they described caregivers as influential, suggesting that when caregivers “*do alcohol or smoke or anything*”, then A-CMCs may be more likely to view these behaviors as normal or appealing. “*I think it’s like your parents are your role models and I think when parents do alcohol or smoke or anything, I think it’s like the children think it’s right to do it because they see their parents doing it*” *(Teen 4)*. Several A-CMCs also found it hypocritical and became frustrated when their parents drank and smoked while discouraging their use. One A-CMC elaborated this point, noting “*it’s like, do what I say not as I do, where they drink and drink and smoke and do all these drugs and stuff and then expect us not to do them, we feel a bit angry and then we’re more inclined to do them*” *(Teen 2)*. In addition, A-CMCs emphasized that exposure to AOD use was common. Some A-CMCs commented that they observed peers engaging in drinking, smoking, vaping, and pill use, while others raised concerns that prescribed “*pills that you’re taking, that you need to take, a lot of the times they can be addictive as well*” *(Teen 2)*, which blurred the line between medical and nonmedical substance use.

Caregivers also discussed awareness of AOD through the lens of risk severity, access, and illness-specific considerations. Caregivers named a wide range of substances (e.g., “*Molly, Oxy, stuff cut with…the really deadly…Fentanyl*” *(Caregiver 3)*), underscoring their awareness of both “common” and high-lethality drugs. “*My kids are now both in college. So, I mean, we’re having these conversations with them regularly. And there’s unfortunately a lot of cocaine use. And a lot of overdoses happening…I mean, there’s almost no drug that they don’t know about.*” *(Caregiver 3)*. Caregivers further emphasized that for A-CMCs, messaging around AOD use is complicated as these youth are told to be highly involved in medication adherence and self-management, while simultaneously being warned about AOD. “*In the case where you’ve got chronically ill kids, it was almost from a very young age…they’re taking medication. And so, we’re constantly drumming into their heads, ‘It’s important to take it, it’s important to take it on time.’ So, you’re having two conversations with them. ‘These are the good drugs. And these are the harmful drugs’*” *(Caregiver 3)*.

Both A-CMCs and caregivers highlighted illness-specific risk, noting that AOD use can interfere with disease management (e.g., “*impact[ing] their seizure control*” for epilepsy, causing “*dangerous*” side effects when mixed with medications) and may have heightened consequences when layered onto existing health vulnerabilities. One caregiver noted that “*…availability of drugs is higher for kids who are in critical care. And the likelihood that they’re going to have painkillers after a procedure is so much higher. So that’s kind of the complicated issue*” *(Caregiver 3)*. Another raised concerns that A-CMCs might not yet be able to navigate these risks independently because “*teenagers…aren’t quite developed enough to use the kind of judgement that adults might*” *(Caregiver 1)*. These descriptions demonstrate that for A-CMCs, substance-related risk is interpreted through family norms, chronic illness self-management, and the blurred boundaries between treatment, dependence, and misuse.

Theme 2: Structural and workflow conditions shaping when and how inpatient SBIRT feels acceptable. This theme focuses on the structural and workflow conditions under which SBIRT is introduced in inpatient care—when it occurs, how questions are delivered, and how much burden it adds to an already demanding hospitalization experience. This theme aligns with HEIF themes of Physical Structures and Economies both of which are within the Societal Context domain, and CFIR Inner Setting domain. A-CMCs and caregivers’ comments in this theme centered less on who asked the questions and more on whether SBIRT fits the realities of inpatient care for A-CMCs.

A-CMCs and caregivers generally anticipated that SBIRT would be appropriate and important in the hospital setting, particularly when it was clearly connected to an A-CMC’s health and care needs. At the same time, both groups emphasized that the way SBIRT will be introduced and delivered may strongly shape whether A-CMCs respond honestly. For instance, A-CMCs supported being asked about AOD use, especially when clinicians “*emphasized confidentiality*” and explained that screening information “*only helps your treatment*” *(Teen 1)* (e.g., understanding risks or interactions with medications) and is not used to judge them or “*what is thought of you as a human being.*” *(Teen 1)*.

However, multiple A-CMCs expressed concerns about the timing of screening, with the first day following admission viewed as an “*overwhelming moment*” when “*there’s a lot going on*” and “*a lot more to process.*” One A-CMC noted that because “*a lot is happening at once, that what the person is saying would make you feel like that would be the time to lie or withhold or distort answers*” *(Teen 3)*. For this reason, several A-CMCs suggested conducting the screening part of SBIRT at least one day after admission or after they were more settled. One A-CMC explained, “*Waiting till you’re kind of more comfortable with the setting and with the doctors and nurses that are coming into the room… but not during the first day or if I’m in the ER then it’s more overwhelming if they ask that question because there’s so much more things going through your head and it’s a lot more to process*” *(Teen 3)*. Participants framed these concerns as questions of SBIRT design and workflow fit within hospitalization rather than opposition to screening itself.

On the other hand, caregivers also appreciated SBIRT and said that knowing that their A-CMC was getting screened would make them feel “*relieved that somebody would be addressing* [AOD use] *in that way, because it’s important*” *(Caregiver 1)*. Caregivers also emphasized the need to “*contextualize*” the purpose of AOD questions for A-CMCs “*within the larger conversation around care*.” One caregiver emphasized that providers can make it explicitly clear why they are asking, how it relates to A-CMCs’ health, and “*how the teen is being trained to care for their own condition” (Caregiver 2).* Caregivers further suggested that the utility of SBIRT would depend on a developmentally appropriate approach that supports privacy via “*separate conversations with young adults or teenagers,*” and “*some level of consent required beforehand*” *(Caregiver 5)*. Another caregiver also expressed that it would be helpful for clinicians to explain why questions are asked, and link screening to health needs “*to create the space where they can answer honestly*” *(Caregiver 2).* This caregiver also raised preferences for alternative modalities (e.g., “*online answering*” or text-based options) that might make disclosure easier, and some suggested that consent or advance preparation could improve comfort and buy-in. Along the same lines, A-CMCs highlighted that protected time with their provider was a concrete resource needed within clinical encounters. A-CMCs described private time as important for feeling comfortable raising sensitive topics and for enabling more candid discussion during screening and counseling. When asked, “how do you feel about having that opportunity to have this private time with your doctors or nurses?”, one A-CMC responded: “*I feel like it’s very important personally*” *(Teen 2)*.

Caregivers framed patient needs and resources more broadly, emphasizing that AOD use can compound other challenges A-CMCs may already be navigating (e.g., health-related stressors, psychosocial vulnerabilities), and that families come “*from different contexts and realities*” *(Caregiver 3)* with substantial variability in background, context, and experiences that could make it difficult to implement SBIRT in a “*one-size-fits-all*” way while still meeting the individual needs of patients. Caregivers also described competing priorities as a key constraint, noting that the utility of addressing risky AOD use may depend on what else the family is managing in the moment (e.g., acute medical concerns, treatment decisions, logistical and emotional burden). When asked, “how important is drug and alcohol use in comparison to other needs A-CMCs and families may have?”, one caregiver said “*The hierarchy of needs is—if they’re trying to find a home, food, those obviously are most important. Depending on what’s going on in a family, or an individual’s life, it’s going to be hard to answer that broadly*” *(Caregiver 1).* “*I think that to the degree it could be a co-traveler with other social influencers of health, right? Things that can compound preexisting vulnerabilities. Whether that’s your condition, or other circumstances, that it’s important just like all the other things are, that we try to help them manage. So I do think it’s important, but I can see where it might not need to be the first thing that’s asked*” *(Caregiver 3).* Together, these accounts indicate that SBIRT acceptability depended not only on the content of screening, but also on whether it was perceived as fitting the A-CMC’s and family’s immediate priorities during hospitalization.

Theme 3: Relational safety: confidentiality, autonomy, and respectful communication within inpatient SBIRT. Theme 3 centers on the HEIF’s Clinical Encounter and Patient Culturally Relevant Factors domains, describing how confidentiality practices, respect for A-CMCs’ autonomy, communication style, and prior experiences of mistrust shaped whether A-CMCs felt safe answering SBIRT questions honestly. Whereas Theme 2 emphasized the structural “when and how” of SBIRT delivery, this theme focuses on the relational and cultural aspects of the encounter itself.

Caregivers and A-CMC described the clinical encounter as a key determinant of whether adolescents felt safe, respected, and willing to disclose sensitive information. They emphasized that trust could be strengthened or undermined by clinicians’ communication style, attention to adolescent autonomy, and the consistency of confidentiality practices. A-CMCs described mixed experiences with confidentiality and private time during hospital encounters. Several A-CMCs described experiences where they felt surprised or “tricked” into disclosing information without fully understanding that it might be communicated to caregivers, which undermined trust. For instance, one A-CMC recalled, “*I remember when I turned 12, they said something really quick and brief like, ‘oh, yeah, yeah, by the way, we can do this thing and you cannot say anything. Okay, bye,’ but what was kind of interesting is that they would trick me. Well, not trick me. It’s kind of hard to explain, but when I—they wanted me to say something and said, ‘this is not confidential, right?’ And since I didn’t exactly know too much of what that word meant, I would just say, ‘yeah, yeah, yeah, you can—this is not confidential and you can I guess share this,’ but I didn’t really know what I was putting myself in and I didn’t know that this information would be shared with my parents and stuff until about a couple years ago*” *(Teen 2).* Another A-CMC shared that “*doctors asking their patients…don’t treat the questionnaires…serious. They’re like, ‘oh, sorry, I gotta get through this’ and that wouldn’t make me feel comfortable talking about it because…you’re just trying to rush through it…And I feel like if they come in with more empathy and talking about it like they care…would have a lot of more patients talk…and not feel as judged*” *(Teen 6).* Together, these narratives show that A-CMCs’ willingness to participate in SBIRT depends on both what is promised about confidentiality and how seriously and empathetically questions are asked.

A-CMCs also described concerns with the tone of screening. Several A-CMCs described standard admission questioning as feeling like an “*interrogation*” and noted that being “*put on the spot*” further increased the “*inclination to lie*” and say “*no, no, no, no, I never,*” particularly when they feared consequences or “*getting in huge and serious trouble*” *(Teen 2)*. The same A-CMC described that this dynamic could trigger “*spiraling*” into a “*panic*” and lead to “*why a lot of kids will not answer that question*” *(Teen 2).* A second A-CMC similarly emphasized that the early hospitalization context, particularly when first arriving to the inpatient unit, contributed to the interrogative feel of screening: “*When you’re admitted and first move up to the floor, they all [ask about] drugs and alcohol. And it’s the way they make your parents leave the room, but that does feel like an interrogation*” *(Teen 1).* These accounts suggest that the sequencing, pace, and bundling of SBIRT questions can make screening feel either supportive or coercive.

A-CMCs also described fear of shame or getting in trouble as a barrier to honesty about AOD use. One A-CMC explained, “*in order for there to be more truth about what we’re telling them, we would want almost confirmation that we wouldn’t be getting shamed for it or yelled at or our parents wouldn’t get in trouble for it if we were to have done something*” *(Teen 3)*. The same A-CMC added, “*Making sure you’re aware that it is confidential and sometimes explaining what that entails just to make sure they know that they’re not going to get yelled at for it by their parents or other doctors or whoever*” *(Teen 3)*. These comments indicate that A-CMCs may enter SBIRT encounters with only a partial and sometimes inaccurate understanding of what confidentiality means, and that clear, developmentally appropriate explanations of confidentiality and its limits are a precondition for honest responses. Furthermore, beyond AOD use, A-CMC perceived that clinical encounters often prioritized physical health concerns and overlooked mental and behavioral health, with one A-CMC noting that hospitals “*fail in most cases to keep me safe or healthy*” because “*most of my problems are not physical and… they’re in fact mental” (Teen 2).* Many A-CMCs described prior experiences feeling “*like I was never heard*” or that their “*worries*” and “*anxieties*” were not believed or “*accounted for*” during their hospital stay. These broader experiences of not feeling heard or believed shaped whether A-CMCs trusted SBIRT questions as genuine invitations to share concerns.

Both A-CMCs and caregivers also identified how confidentiality procedures are operationalized as an important part of autonomy and emotional safety. Both described SBIRT delivery in the inpatient setting as complex primarily because of confidentiality procedures, caregiver presence, and the practical realities of how screening is administered. Furthermore, both viewed privacy practices as essential but sometimes inconsistently implemented or difficult to navigate. A-CMCs frequently noted that clinicians ask caregivers to step out, but some believed that it put them “*in an uncomfortable situation,*” particularly when providers ask them, in front of the parent, whether they want their caregiver to leave. One A-CMC described this as feeling awkward or pressuring because it places responsibility on the patient and can create tension in the room. “*I feel that it puts us in an uncomfortable situation if the doctors ask if you want your parents to leave or not. And then once you’re done it’s gonna be awkward. They’re like, why did you say to leave and stuff like that*” *(Teen 4)*. This A-CMC also shared that “*I think the talking to your nurse or your doctor, I think it’s helpful. But your parents I think are also nosy knowing that you’re going to talk about stuff without them…So, I think they’re really nosy and like also ask about it. And so it’s awkward*” *(Teen 4)*. A-CMCs preferred that clinicians take the lead by directly asking parents to step out, rather than positioning it as the adolescent’s choice in front of the caregiver. One A-CMC specifically noted that “*If you (teen) ask them to leave, they’ll be like, why’d you ask me to leave? What are you doing? But if you—if the doctor is saying like, hey, parents, it’s time to leave, then really in that case it’s much safer and much better for the teen in the instance*” *(Teen 2).* “*When it’s like, ‘do you want them to’ versus ‘it’s time’…for some people it could make all the difference*” *(Teen 1),* because the latter would yield far more honest reporting. A-CMC also raised logistical concerns about screening platforms, noting that certain modalities (e.g., visible screens in shared spaces) do not feel private and may inhibit honest responding. Several A-CMC supported the idea of “*on paper questions*” because it “*wouldn’t be as likely for [caregivers] to be watching over you and seeing what you are writing*.” One A-CMC recommended a hybrid or “*mixed*” approach that combines private written questions with a follow-up “*conversation with confidentiality*,” rather than relying on a single platform. Several A-CMCs described rarely hearing confidentiality discussed explicitly and expressed uncertainty about what information is shared with caregivers. One noted, “*Confidentiality is, at least in my understanding and my experience, that it’s when we are legally obligated not to tell what you have said unless you are going to hurt yourself or others…But basically that if you’re hurting yourself or if you’re hurt or someone else is hurt, technically they do have by law have to tell somebody, but if none of those two things aren’t happening by law, they usually should not be telling anybody else*” *(Teen 2)*. A-CMCs may enter SBIRT encounters with only a partial and sometimes inaccurate understanding of what “confidential” means, which could influence their comfort disclosing AOD use. These comments underscore the need for explicit, developmentally appropriate explanations of confidentiality at the outset of SBIRT.

Caregivers highlighted variability in clinician approach and a perception that the encounter is shaped by broader constraints in the health system. Caregivers generally accepted being “*asked to step out*” so clinicians could ask A-CMCs about AOD use. However, caregivers emphasized that provider communication differed widely with some clinicians being perceived as more “empathetic” or “*receptive to conversation than others*”, while others felt more “*business-like*,” “*curt*” or rushed. One caregiver noted that physicians do not always have the time to go deeply into concerns or provide thorough explanations, and sometimes appear uncertain about the next steps, resulting in referrals elsewhere that feel like an added burden. In this context, adding SBIRT was described as one more step layered onto already fragmented care. “*I was going to say there has been times at other clinics where sometimes they don’t know what to do with my child. And it’s like, we’re told to first go to see the pediatrician before you go to the emergency room. And it’s like it’s an extra step, where we end up at the emergency room*” *(Caregiver 4)*. Another caregiver raised concerns that clinical structures often do not adequately serve youth with disabilities or complex needs because their “*needs are not built into the structures of the processes, the physical place*”, creating frustration with how disability is handled and the limited support navigating practical issues (e.g., insurance and medication approvals). Thus, in their view, for SBIRT to feel worthwhile, it would need to be paired with accommodations and streamlined pathways to help, rather than additional disconnected referrals.

When encounters went well, A-CMCs emphasized clinicians who took time, listened directly to them (not only their caregivers), and made space for their voice. Several A-CMCs lauded hospital staff who were genuine or “human” and able to “make jokes” without being “super, super serious.” One A-CMC specifically praised the child life specialist for helping them feel comfortable, informed, and advocated for. “*She asked me what would be helpful to make sure I was feeling heard throughout the stay…One of the things… was making sure that just everything’s straightforward and not sugarcoated…They made sure I understood it and they weren’t hiding anything from me*” *(Teen 3)*. Another A-CMC also pointed to visible cues in the environment as meaningful, noting that signage reflecting diversity, equity, inclusion, and accessibility can signal whether the setting feels welcoming or aligned with patients’ identities and values. “*I feel like that’s respected…I see some signs. I see them in some places. I see them less in other places*” *(Teen 2). A-CMCs suggested that these* environmental cues influenced whether they perceived the hospital as a safe place to disclose sensitive information, including substance use, during confidential SBIRT conversations. A few caregivers portrayed the clinical encounter as influenced by both their sense of “*trusting in the provider*” and “*trusting in the culture*,” noting that systemic pressures constrain responsiveness because “*systems are hard to change*” *(Caregiver 3).* This distinction between trust in individual clinicians and mistrust in the broader hospital culture informed caregivers’ expectations about whether SBIRT-identified concerns would actually be addressed.

Across both focus groups, participants emphasized that A-CMCs’ willingness to engage honestly in SBIRT is grounded in relational safety at both the interpersonal and community levels. One A-CMC explained that “*I expect to be treated with respect and I, most of all, I expect not to be treated like a basket case… I just expect to be treated like a human being*” *(Teen 2)*. A caregiver described that “*As a parent, like, I would want my child to have trust and be able to address all of the answers honestly*” *(Caregiver 4)*. For the individual A-CMCs and caregivers in our sample who described racially or ethnically minoritized identities or visible disabilities, “*being treated like a human being*” referenced not only interpersonal warmth, but also freedom from stereotypes, pity, or assumptions about their competence, as with the equity-relevant observations above, these are individual accounts rather than subgroup-level findings. A-CMCs described valuing clinician communication that is warm, nonjudgmental, and developmentally attuned, noting that lightness or humor can make sensitive discussions feel less intimidating. “*I feel like sometimes…kind of like making jokes and stuff with them. It’s actually like a conversation and not super, super serious. Even when sometimes you’re talking about super, super serious topics because then it can just be overwhelming. You don’t even know what to say to them about it. Yeah, because that makes it feel less overwhelming…or they’re more human*” *(Teen 1)*. Several adolescents linked this sense of “human” interaction to feeling seen beyond their diagnosis, age, or racial/ethnic identity, and described SBIRT conversations as more acceptable when providers first signaled respect for their broader lived experience.

Both A-CMCs and caregivers highlighted that trust is influenced not only by individual clinicians but also by the broader system, including continuity of relationships, perceived follow-through, and whether the hospital culture feels responsive to families’ concerns. A subset of individual caregivers—some of whom described their A-CMC as racially or ethnically minoritized, and some of whom described managing complex disabilities—emphasized that “textbook” expectations often did not match their children’s realities. They suggested that culturally relevant SBIRT would benefit from inviting family narratives about culture, neighborhood context, and disability rather than relying solely on standardized questions. One caregiver noted that “*You want them to really, really listen to our experience, and what we’re seeing outside of the office, and see if that differs from what they might have expected, or what is the textbook case. And I want them to be empathetic, and empathetic to that experience, as well*” *(Caregiver 1).* Participants tied system level trust to community histories of care at this hospital; for example, prior negative experiences with staff not believing pain reports or delaying tests were seen as part of broader patterns affecting “*families like ours*,” which shaped expectations about whether substance-use disclosure in SBIRT would actually lead to help. Caregivers noted that negative experiences can erode trust and circulate through family and community networks, reinforcing the importance of consistent, relationship-centered care. “*So, originally, with the first day that I had I went in there thinking I can trust them. They’re doctors, they tell the truth. But then things kind of went backwards when I was in the ER, and we found out one of the nurses did not tell the truth about labs so that kind of made it harder for me throughout that stay to know whether I can trust a person or not trust a person*” *(Teen 3)*. Together, these experiences underscore that SBIRT will feel trustworthy when it is embedded in relationship-centered care that acknowledges families’ histories with the hospital and actively works to repair and sustain trust and trustworthiness over time.

Theme 4: Structural barriers and enablers shaping SBIRT follow-up. Theme 4 focuses on the structural conditions that influence whether the brief intervention and referral-to-treatment components of SBIRT can be carried out after risk is identified, reflecting HEIF Societal Context constructs related to Physical Structures and Economies and CFIR Outer Setting constructs related to patient needs and available resources.

Participants described how the physical and material conditions of care such as the hospital environment, transportation demands, and resource availability, shape families’ experiences of hospitalization and their capacity to participate in SBIRT-related screening and follow-up services. Specifically, one A-CMC emphasized that the hospital environment can carry emotional weight and noted “*it can be very overwhelming to just even be in the building*” *(Teen 1),* particularly when prior experiences in that setting are associated with distress or difficult medical events. A-CMCs suggested that when being in the hospital feels overwhelming, they have less energy and attention for additional questions about AOD use, which may reduce openness to SBIRT during certain visits. In addition, one A-CMC described the hospital’s location and the transportation demands as causing fatigue and strain that reduces openness to screening. “*For me, it’s a little difficult because… I live 30 min out, but with the traffic, it’s like it takes a while…So…when I know I have to go to the hospital, I know it’s gonna take up most of my day*” *(Teen 6)*. For A-CMCs, long travel times and all-day visits made it harder to engage in additional SBIRT screening or attend recommended follow-up appointments beyond the immediate reason for hospitalization.

Similarly, some caregivers described the financial and physical burden of repeated hospital visits, including parking costs, limited parking availability, and travel challenges, especially when families make multiple trips or cannot coordinate appointments on the same day. One caregiver shared that “*I think one of the biggest barriers is parking. Even if you have a car, and you can get there, there’s very little street parking. If you can find a street spot, it’s pretty pricey meters. And then even with the discount, if you’re a frequent flier it really adds up*” *(Caregiver 1)*. Another explained that “*It’s just not an easily accessible location. It takes a long time to get there from wherever you are, unless you live right down there in that neighborhood. And most people don’t. And so if you’re coming from any distance, you have to deal with the highway or a long multiple-stage public transportation relay…there’s a lot of contingencies for you to even be able to get there*” *(Caregiver 3)*. This caregiver also highlighted gaps in downstream capacity and access that would create challenges with the referral to treatment component of SBIRT, expressing concern that families may be identified and referred for follow-up services but then face inadequate mental and behavioral health resources (e.g., limited availability, long waitlists, understaffing). “*Everyone knows, if we have to see psych, and god forbid you’re a new patient, they are understaffed to the hilt…So I’m wondering what the follow-through is to make sure that people are actually able to get a provider connected with them, in a timely fashion. Along the lines of substance abuse, if it’s an emergency, and it’s impatient, they’re probably going to be tended to in the hospital. But follow up with that, and you’re not going to see a provider for three, or four, or five more months. How is that being dealt with? So that’s kind of my biggest concern, is that we notify on the problem, we get them connected, the provider says that they’re going to do something, but then there’s actually not resources to follow up with it on the back end*” *(Caregiver 3)*. Together, these accounts suggest that SBIRT can feel like “*one more appointment*” or “*one more thing*” layered onto already costly and exhausting visits, which may reduce families’ willingness to add separate screening or follow-up encounters focused on substance use. Additionally, SBIRT that flags risk without realistic, timely access to treatment may erode trust in the process. Thus, SBIRT implementation for A-CMC may need to be designed to minimize added visits and out-of-pocket costs and ensure that any recommended follow-up is feasible within families’ transportation and financial constraints.

Across all four themes, no participant expressed outright disagreement with the premise of inpatient SBIRT or described it as inappropriate for A-CMCs. Variation among participants centered on how screening and follow-up should be implemented rather than whether it should occur. Given the small, self-selected sample, this convergence should not be interpreted as evidence that inpatient SBIRT is universally acceptable to A-CMCs and caregivers, but it does suggest that, among participants willing to discuss these topics, acceptability concerns were more often about implementation design than about the intervention’s underlying rationale.

## 3. Discussion

This exploratory qualitative study examined caregiver and A-CMC perspectives on the acceptability, feasibility, and equitable implementation of inpatient SBIRT in the context of hospitalization for chronic medical conditions. Throughout this discussion, we maintain a distinction between participants’ lived healthcare experiences, including confidentiality practices, trust, and communication style, which reflect direct prior encounters, and their hypothetical or anticipatory responses to a proposed inpatient SBIRT program, which they had not yet experienced. Because participants were asked to consider a proposed inpatient SBIRT approach rather than reflect on receipt of an established SBIRT program, the findings should be interpreted as anticipatory, participant-informed perspectives that can guide future co-design and implementation research, not as an evaluation of an existing intervention. Participants described how sociopolitical context, clinical encounters, and structural conditions may shape both willingness to disclose AOD use and the perceived feasibility and acceptability of SBIRT in hospital settings. Applying the CFIR and HEIF together extends prior SBIRT work by describing how confidentiality practices, stigma, and structural barriers intersect specifically for hospitalized A-CMCs and their families, a population often underrepresented in implementation studies [38,39]. Given the small, single-site sample and focus group design, these findings are intended to describe participant-identified determinants of acceptability and feasibility, generate hypotheses about contextual factors and identify potential adaptations, rather than establish the prevalence, relative importance, or effectiveness of specific SBIRT implementation strategies. These participant-identified determinants including chronic illness management, disability, and long-standing inequities in how youth and families experience hospital care align with epidemiologic evidence that peer norms and social environments contribute to adolescent risk behaviors and undermine help-seeking when illness or vulnerability is stigmatized [6,40,41]. In turn, they point to promising equity-oriented adaptations such as tailored confidentiality workflows and relational practices for A-CMCs that may inform future co-design and implementation research.

Interpreted through the CFIR, A-CMCs’ concerns about timing, tone, and modality map onto key Intervention Characteristics (e.g., complexity, design quality and packaging) and Outer Setting domains (e.g., patient needs and resources, available resources), while caregivers’ emphasis on follow-up pathways underscores the importance of implementation climate and structural support. The HEIF further draws attention to sociopolitical forces and patient culturally relevant factors, including stigma, ability status, and prior experiences of not being believed, which shape whether SBIRT is perceived as supportive or risky. In the context of pediatric inpatient care for A-CMCs, where youth routinely depend on the health system for survival and symptom control, these dynamics have particular salience [26,27]. SBIRT is judged not in isolation, but against a backdrop of cumulative encounters that may have affirmed or undermined families’ trust and the trustworthiness of the hospital system. Taken together, these frameworks clarify that barriers and facilitators to A-CMCs’ AOD disclosure are not solely individual or attitudinal but embedded in institutional structures and relationships [42,43].

The combined application of the CFIR and HEIF also suggests a refinement to how inpatient SBIRT determinants are conceptualized. In this setting, intervention characteristics such as timing, modality, and workflow complexity were not experienced as merely technical design features. Instead, participants linked them to confidentiality, autonomy, and whether disclosure felt emotionally safe. The CFIR helped organize workflow and setting determinants, whereas the HEIF made visible how disability, institutional trust, sociopolitical context, and prior inequitable care shape their meaning and consequences. These findings suggest that relational safety and equity may function as cross-cutting conditions that influence the feasibility and acceptability of multiple implementation determinants rather than as discrete contextual considerations.

Sociopolitical context and AOD perceptions. Consistent with prior research on adolescent AOD use, A-CMCs and caregivers in this study situated AOD use within broader sociopolitical and cultural contexts, describing how peer norms, media exposure, and stigma shape both perceived acceptability of use and willingness to seek help [41,44,45]. Our findings corroborate prior research demonstrating that casual or experimental use is often normalized or even glorified, while “having a problem” is heavily stigmatized, a distinction that A-CMCs perceived as discouraging honest disclosure and formal help-seeking [46]. Caregivers similarly noted ubiquitous access and social messaging that may shape risk environments which has been found in the literature [44,47]. At the same time, this study extends existing work by showing how A-CMCs and caregivers interpret these norms through the lens of chronic illness management and medication use, including blurred boundaries between prescribed and nonmedical substances and heightened concern about illness-specific risks (e.g., seizure risk in epilepsy) [9]. For A-CMCs whose daily routines already center around medications, hospitalizations, and complex regimens, “drugs” are not an abstract category but part of survival, which complicates traditional prevention messages. These nuances suggest that SBIRT for A-CMCs may benefit from explicitly addressing medication by highlighting the AOD interactions and the mixed messages that youth receive about “good” versus “bad” substances, rather than treating AOD use as separate from chronic condition management. Doing so may be particularly important for A-CMCs from communities that already experience inequitable pain management and differential prescribing, for whom messages about misuse can feel especially fraught.

Acceptability of SBIRT and Conditions for Disclosure. Across participants, SBIRT was seen as an appropriate and potentially valuable model for inpatient settings, particularly when framed in terms of supporting A-CMCs’ health needs rather than judgment or punishment. This aligns with recommendations that universal AOD screening be integrated into routine youth healthcare to facilitate early identification and intervention [17]. However, our data highlight how delivery details shape acceptability for hospitalized A-CMCs. Adolescents voiced concerns about the timing and tone of screening, describing admission and other high stress moments as overwhelming and “interrogative,” which increased the likelihood of withholding or distorting answers. This echoes broader literature suggesting that clinician communication style and the perceived purpose of screening significantly influence adolescent disclosure in clinical settings [28,48,49] and they underscore the importance of aligning SBIRT workflows with the realities of inpatient care. For A-CMCs, who often arrive at the hospital exhausted, in pain, or anxious about procedures, even well-intentioned screening can be experienced as one more demand unless it is carefully sequenced and clearly linked to their immediate health concerns.

Caregivers similarly endorsed SBIRT and described relief that clinicians were addressing AOD in the context of existing vulnerabilities, yet they emphasized that A-CMCs’ honesty depends on developmentally appropriate approaches that clearly explain why questions are being asked and how information will inform care. For A-CMCs in particular, caregivers noted that AOD screening may need to be integrated into conversations about medication adherence and illness management, rather than added as a disconnected checklist item. They also highlighted that for A-CMCs from racially and ethnically minoritized groups and those with disabilities, SBIRT may be interpreted through prior experiences of bias in pain assessment, behavioral labeling, or disciplinary responses, amplifying the need for explicitly nonjudgmental framing. These perspectives reinforce the CFIR’s emphasis on intervention coherence and fit with existing clinical practices [32] and support tailoring SBIRT messaging to highlight its relevance to each A-CMCs’ condition and treatment plan.

Confidentiality, Trust, and Relational Safety. A dominant theme emerging from our data was the centrality of confidentiality, trust, and relational safety to authentic adolescent disclosure [41]. A-CMCs in our focus groups underscored that uncertainty about how information would be used, or whether it would be shared with caregivers, may undermine other A-CMCs’ willingness to answer honestly, consistent with studies showing that confidentiality assurances are strongly associated with increased adolescent disclosure around sensitive topics such as substance use, sexual behavior, and mental health concerns [50,51]. Because AOD use screening and brief intervention typically occur within the same clinical exchange, A-CMCs’ relational safety and confidentiality concerns speak not only to whether A-CMCs feel able to disclose AOD use, but to whether the subsequent brief intervention conversation itself feels safe, respectful, and non-punitive. Clinician communication style, developmental attunement, and trust, both in the individual provider and in the broader hospital culture, therefore emerged as determinants of the brief intervention component of SBIRT, not solely of the initial screening interaction. For instance, several A-CMCs described examples during previous hospitalization of feeling “tricked” when confidentiality explanations were incomplete or when information was shared with caregivers in unanticipated ways, suggesting that poorly implemented privacy procedures may have iatrogenic effects on trust. A-CMCs’ preference for private time with clinicians and clear explanations of confidentiality aligns with clinical practice recommendations that suggest routine provision of confidential care and explicit dialog about privacy protections to facilitate accurate reporting [52,53]. In pediatric inpatient units, where parents are often continuously present and deeply involved in care, establishing this confidential space may require intentional, standardized (but malleable) workflows so that requests for privacy are not placed on A-CMCs themselves or interpreted by families as signs of wrongdoing. And rather than simply adding more screening questions, health systems may benefit from ensuring that confidentiality practices are transparent, consistently applied, and developmentally attuned to avoid eroding trust in encounters intended to support A-CMCs.

Caregivers mirrored this emphasis on trust, highlighting that confidentiality practices could be paired with empathy, continuity, and a broader culture of responsiveness to A-CMCs’ concerns [26,54]. When clinicians are perceived as rushed, dismissive, or constrained by systemic pressures, both A-CMCs and caregivers reported feeling less confident that disclosure would lead to meaningful support. These dynamics were particularly salient for caregivers of racially and ethnically minoritized A-CMCs and for those managing complex disabilities, who described “textbook” expectations that did not match their teenagers’ realities and cumulative experiences of not being believed. These perspectives align with the HEIF’s Clinical Encounter and Patient Culturally Relevant Factors domains and suggest that SBIRT implementation should be embedded within broader efforts to strengthen relationship centered care for A-CMCs, including explicit attention to disability, mental health, and prior experiences of not being heard.

Delivery Challenges and Workflow Considerations. Participants highlighted several implementation complexities that could impede consistent delivery of SBIRT in hospital settings. Both A-CMCs and caregivers described digital platforms used during hospitalization as double-edged. While some electronic formats were seen as potentially reducing face-to-face pressure, others (e.g., large, room-visible screens) were experienced as conspicuously non-confidential, raising concerns about who might see responses. These accounts add nuance to the growing literature on digital screening by demonstrating that the perceived privacy and trustworthiness of specific platforms may influence disclosure more than the mere presence of technology [55].

Participants frequently proposed hybrid models such as brief private written or electronic questions paired with supportive clinician conversations, as more acceptable than relying on any single modality. This aligns with the CFIR’s focus on design quality and packaging, underscoring that SBIRT tools should augment, rather than replace, relational care [17,31]. Caregivers also emphasized structural barriers to translating screening into sustained engagement or referral, including transportation and parking burdens, difficulty coordinating appointments, and limited capacity in downstream mental and behavioral health services. These concerns resonate with the HEIF’s emphasis on physical structures and sociopolitical forces, suggesting that even well-implemented screening will have limited impact if follow-up services remain inaccessible or under-resourced [34]. For medically complex youth who already experience disproportionate financial and logistical strain from frequent hospitalizations, SBIRT that identifies AOD use risk without feasible pathways to care may risk widening, rather than closing, equity gaps.

Exploratory Implementation Considerations and Future Research Priorities. Although these findings do not establish best practices or policy recommendations, they identify several participant-informed considerations that warrant co-design and evaluation in future inpatient SBIRT implementation studies. First, future inpatient SBIRT initiatives could examine the feasibility and acceptability of incorporating structured confidentiality scripts and procedures that normalize private adolescent–clinician interactions and clearly delineate what information will and will not be shared [53]. As a candidate adaptation requiring future testing, scripts and training could be piloted to address how to create private time in rooms where caregivers are usually present, with the approach that clinicians—not A-CMCs—take responsibility for asking caregivers to step out. Whether this specific approach improves disclosure or trust remains an empirical question for future co-design work. Second, training for clinicians could foreground communication strategies that balance sensitivity with clarity, particularly in the context of complex pediatric medical care where emotional burden is high [9,27]. Third, digital screening tools and workflows could be evaluated not just for feasibility but for perceptions of privacy and trustworthiness among A-CMCs, as these perceptions may influence disclosure more than the medium itself [55]. These findings raise questions for policy and health system leaders about how to reconcile confidentiality protections with caregiver engagement in a way that promotes adolescent autonomy while maintaining family involvement when appropriate, as supported by family-inclusive SBIRT adaptations in the literature [56].

Based on the consistency and strength of support across both focus groups, we prioritize three candidate strategies for co-design and empirical testing in inpatient SBIRT for A-CMCs: (1) standardized, equity-oriented confidentiality workflows that normalize private adolescent–clinician interactions, which was the most consistently and strongly supported adaptation across both A-CMCs and caregivers; (2) flexible screening timing that accounts for acute clinical demands, which was strongly and specifically supported by adolescent accounts; and (3) accessible, family-inclusive follow-up pathways, which was strongly supported by caregiver accounts of transportation, cost, and referral-capacity barriers. Digital modality preferences and clinician communication training, while also raised by participants, are treated here as secondary considerations to be incorporated within these three priorities rather than as co-equal strategies. Future studies should examine the feasibility, acceptability, equity implications, and effects of these adaptations across diverse pediatric inpatient settings. Such work could assess not only screening and referral completion, but also adolescents’ perceived relational safety, disclosure, access to follow-up care, and whether these outcomes differ across racial, ethnic, disability, gender, and socioeconomic groups. It may be beneficial for these procedures to be introduced early, revisited over time, and adapted to developmental level and disability status to avoid misunderstandings that may damage trust. Furthermore, future SBIRT studies could align screening timing and modality with clinical realities. Rather than introducing detailed AOD questions during the most acute phases of admission, when A-CMCs are overwhelmed and disclosure may be least reliable, teams could prioritize screening once families are more settled, unless information is immediately needed to guide medications or acute management. In parallel, health systems could co-design and test digital tools with A-CMCs and caregivers to ensure that formats feel genuinely private (e.g., personal devices, discreet interfaces) and that visible shared-screen platforms are avoided for sensitive content [55,57,58]. It is important for future evaluation to incorporate equity-relevant considerations—assessing not only overall screening and referral rates, but whether A-CMCs from marginalized racial, ethnic, disability, and socioeconomic groups are equally likely to have concerns identified and to receive timely, acceptable follow-up care.

Structural and Organizational Considerations for Implementation. The feasibility and equity implications of these promising adaptations will likely vary across healthcare settings. Hospitals differ substantially in behavioral health staffing, referral networks, digital infrastructure, reimbursement mechanisms, and capacity to absorb new screening and follow-up activities. For example, a well-resourced academic children’s hospital may be better positioned to integrate confidential electronic screening, embedded behavioral-health consultation, and telehealth follow-up than hospitals serving rural, under-resourced, or geographically dispersed communities [22]. Similarly, settings with limited specialty treatment availability, transportation barriers, inadequate broadband access, or high levels of uninsurance or underinsurance may have greater difficulty translating identification of AOD-related concerns into timely and acceptable services [20,24,44]. Thus, implementation efforts should assess whether these proposed workflows expand equitable access to confidential, responsive care or inadvertently widen inequities by identifying needs that organizations lack the capacity to address. This reinforces the need for AOD-related and chronic illness care to be coordinated and delivered in parallel rather than sequenced, which has direct staffing and referral-timing implications for organizations planning inpatient SBIRT [59].

Operationalizing participant-informed SBIRT adaptations also requires attention to organizational readiness and resource allocation. Potential requirements include protected time for staff training; role-specific preparation in A-CMCs’ AOD use, confidentiality, brief intervention, and referral procedures; clearly defined responsibility for screening, response to positive screens, documentation, and follow-up; and EHR functionality that supports confidential data capture while accounting for caregiver proxy access and other privacy considerations. Organizations may also need behavioral health, social work, informatics, and data analytics capacity to establish referral pathways, monitor workflow performance, and examine equitable reach and outcomes. These activities may be challenging in the context of competing screening demands, staff shortages, high patient acuity, and uncertain reimbursement for SBIRT services [22,23,24]. Future implementation research should therefore test contextually tailored models, including phased pilots, across varied pediatric inpatient units and settings and evaluate both implementation outcomes and resource implications. These considerations are consistent with findings from a recent qualitative study of pediatric hospital professionals, which identified staffing capacity, role clarity, training, EHR integration, referral availability, funding, and leadership support as key determinants of SBIRT implementation in inpatient pediatric settings [20]. Read together, these two studies offer complementary vantage points on SBIRT implementation for A-CMCs. The present study centers A-CMC and caregiver priorities for how SBIRT is experienced and delivered, while the pediatric hospital staff paper centers the feasibility and organizational determinants perceived by the staff responsible for delivering it [20]. Furthermore, given the fragmented care, transportation burden, and limited behavioral health access our participants described, findings suggest that referral to external services alone may be insufficient to translate SBIRT-identified concerns into completed treatment for A-CMCs. Integrated models of care in which AOD use treatment is embedded within or closely coordinated with existing chronic disease management and behavioral health services, rather than delivered solely through referral to a separate external provider, may better address these barriers. This is consistent with evidence from a randomized comparison of treatment delivery models for adolescents with substance use and co-occurring conditions, which found that concurrently delivered treatment—whether integrated within a single care team or delivered in parallel by coordinated providers—produced better adherence and global outcomes than approaches that addressed one condition while leaving the other unaddressed [59]. For A-CMCs, whose chronic illness management cannot be paused or deferred, this suggests that any AOD-related concern identified through inpatient SBIRT should be met with a referral or intervention pathway designed to proceed in parallel with, rather than sequentially after, ongoing subspecialty and chronic disease care. This may reduce the burden of additional appointments and build on the continuity and trust participants identified as central to feeling safe engaging with SBIRT.

Limitations and Future Research. This exploratory qualitative study has several limitations. The small sample, comprising 13 participants across two focus groups, was designed to elicit in-depth and contextually situated perspectives rather than to estimate the prevalence, relative salience, or distribution of identified themes. Accordingly, the findings should be interpreted as participant-informed, hypothesis-generating insights and not as statistically representative estimates or universally applicable recommendations for inpatient SBIRT implementation for A-CMCs. The findings are contextually bound to a single children’s hospital and to A-CMCs and caregivers who participated in one focus group each and thus may not fully capture the perspectives of A-CMCs and families in other settings or cultural contexts. Transferability may also be limited because participants’ perspectives may be shaped by the institutional culture, clinical services, behavioral health infrastructure, and regional context of this particular setting. These conditions may differ meaningfully in hospitals with fewer specialty resources, and settings serving populations with different racial, ethnic, linguistic, socioeconomic, disability, and geographic experiences. Furthermore, all focus groups were conducted in English, and the findings may not reflect the experiences of A-CMCs and caregivers who prefer languages other than English or who encounter language-access barriers in healthcare. In addition, families willing and able to participate in a virtual focus group may differ from those who did not participate, including their availability, digital access, comfort discussing AOD use, healthcare experiences, or trust in the health system. Moreover, although A-CMCs in our sample represented diverse racial, ethnic, and gender identities, we did not enroll any cisgender A-CMC boys. As a result, the perspectives of boys who identify as male are underrepresented, and our findings may not capture gender-specific norms and concerns that shape AOD use disclosure for this group. The cross-sectional design also precludes assessment of how perceptions of SBIRT evolve over time or after exposure to specific implementation strategies. Future multi-site mixed-methods research could test discrete workflow adaptations identified here and examine their impact on disclosure rates, referral uptake, and perceived relational safety among diverse A-CMC populations. Further work should also investigate how sociopolitical forces and structural barriers differentially shape SBIRT experiences for A-CMCs across racial, ethnic, gender, and socioeconomic groups, and how implementation strategies can be tailored to advance, rather than inadvertently undermine, health equity in pediatric inpatient care. Such work could examine which themes are transferable, which are setting-specific, and how local structural conditions shape the feasibility, acceptability, equity, and outcomes of participant-informed SBIRT adaptations. In addition, A-CMCs and caregivers were recruited as independent, non-dyadic samples. Separate focus groups may have supported candid discussion of privacy, disclosure, and caregiver involvement; however, the design does not permit examination of how these issues are negotiated within specific families or how A-CMC and caregiver perspectives align or diverge in real time. Future dyadic or family-based research could examine how confidentiality, private clinician–adolescent conversations, and follow-up decisions are discussed and managed within families. Finally, because universal inpatient SBIRT was not implemented for A-CMCs at the hospital during the study period, the SBIRT-specific findings reflect hypothetical or anticipatory perspectives rather than direct experience with routine SBIRT. Participants were also discouraged from disclosing personal AOD use experiences. Although this approach supported privacy and allowed the discussion to focus on implementation preferences, future research should examine how these anticipatory perspectives compare with the experiences of A-CMCs and caregivers after SBIRT is implemented in routine inpatient care.

## 4. Conclusions

This exploratory qualitative study identified A-CMCs and caregiver concerns and preferences that may shape the acceptability and equitable implementation of a proposed inpatient SBIRT approach for A-CMCs. The findings should be interpreted as participant-informed, hypothesis-generating insights rather than evidence of best practices or effectiveness. Future multi-site co-design and mixed-methods studies should examine the feasibility, equity implications, and outcomes of the identified potential adaptations across diverse pediatric inpatient settings.

## Figures and Tables

**Table 1:** Demographics of Focus Group Participants.

Demographics	Caregivers (*n* = 6)	A-CMCs (*n* = 7)
Racial Identity		
Black or African American	2 (33%)	1 (14%)
White	4 (67%)	4 (57%)
Something Else	0 (0%)	2 (29%)
Ethnicity		
Hispanic or Latino	1 (17%)	3 (43%)
Not Hispanic or Latino	5 (83%)	4 (57%)
Age (Caregivers)		
18–29		
30–39	1 (17%)	
40–49	3 (50%)	
50+	2 (33%)	
Age (A-CMCs)		
12–13		2 (29%)
14–15		3 (43%)
16–17		1 (14%)
18		1 (14%)
Grade		
6th–8th		3 (43%)
9th–12th		4 (57%)
Gender Identity		
Male	1 (17%)	0 (0%)
Female	5 (83%)	5 (71%)
Trans-male		1 (14%)
Non-Binary/Expansive		1 (14%)

**Figure A1:**
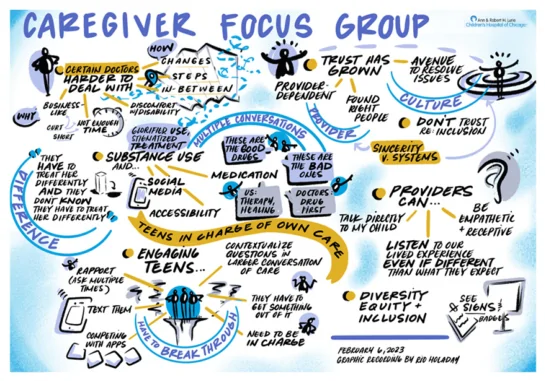
Graphic Recording Picture—Caregivers.

**Figure A2:**
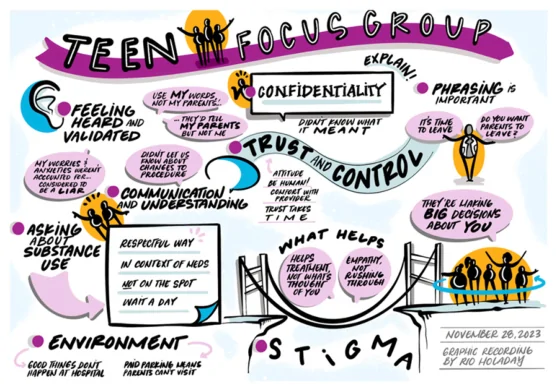
Graphic Recording Picture—A-CMCs.

## Data Availability

The qualitative focus group data generated and analyzed in this study are not publicly available due to the sensitive nature of the transcripts and the risk of compromising participant confidentiality, particularly given the small sample of adolescents with chronic medical conditions and their caregivers. De-identified excerpts relevant to the findings are included in the article. Additional de-identified data may be made available from the corresponding author on reasonable request and subject to Institutional Review Board approval and data use agreements.

## Abbreviations

The following abbreviations are used in this manuscript:

A-CMCs	Adolescents with chronic medical conditions
AOD	Alcohol and other drug
CFIR	Consolidated Framework for Implementation Research
HEIF	Health Equity Implementation Framework
SBIRT	Screening, brief intervention, and referral to treatment

## Appendix A

### Appendix A.1

**Table A1:** Patient and Caregiver Codebook.

Clinical Interaction	Definition: This domain is centered on how the patient and provider choose, adapt, and coordinate the conversation to achieve their shared and personal goals concerning health related matters. Inclusion Criteria: Statements about provider communication quality, type, and/or length; provider interpersonal behavior (e.g., looking at the computer); linguistic competency; availability and/or quality of interpreter; cultural competency (e.g., microaggression, perceived discrimination, knowledge of cultural group); knowledge and skills conveyed or demonstrated about health problem. Include statements that discuss FEELINGS about interactions that happened between provider and patient; including verbal, non-verbal, and nonface-to-face interactions, e.g., messages over MyChart, email, and other forms of communication. Include statements about issues or problems experienced when interacting with a provider and level of understanding regarding patient’s needs. Exclusion Criteria: Exclude statements that do not describe patient–provider interactions, e.g., describing the patient’s state. Do not include descriptions of what someone would like or expect in an interaction and instead code that as Patient Culturally Relevant Factors.
Knowledge & Beliefs about alcohol and other drugs (AOD)	Definition: Patient and caregivers’ attitudes toward and value placed on AOD as an issue for them/their person. Inclusion Criteria: Statements related to understanding the risk factors of adolescent AOD use. Discussion of how substance use impacts them/their person. Statements related to alcohol and other drug use among patients with a chronic medical condition (CMC). Exclusion Criteria: Statements related to familiarity with evidence about the SBIRT and code to Evidence Strength & Quality.
Knowledge & Beliefs about SBIRT-A	Definition: Patient and caregivers’ attitudes toward and value placed on SBIRT, as well as familiarity with facts, truths, and principles related to SBIRT-A. Inclusion Criteria: Statements related to prior knowledge about SBIRT. Statements regarding SBIRT pros/benefits and cons/concerns. Feelings about appropriateness of using SBIRT in their specific setting. Include statements regarding importance of addressing issues related to implementing SBIRT-A such as use of drugs among adolescents, knowledge of adolescent drug use and its impact on them, and knowledge about SBIRT-A. Exclusion Criteria: Statements related to familiarity with evidence about the SBIRT and code to Evidence Strength & Quality.
Intervention Participants	Definition: Strategies for engaging individuals served by Lurie to participate in SBIRT-A. Inclusion Criteria: Include statements related to engagement strategies and outcomes, e.g., how SBIRT-A participants became engaged with SBIRT-A, ideas on informing participants about intervention, apps/networks that may be used to share health information with the participants. Discussion can include the following: - Ways of communicating information. - Timing of introducing SBIRT. - Length of SBIRT. - A-CMC and caregiver reactions to logistics of SBIRT - Technology that may be used to present/complete SBIRT (MyChart, other apps/networks, etc.). Exclusion Criteria: Exclude statements demonstrating (lack of) awareness of the needs and resources of those served by Lurie and whether or not that awareness influenced the implementation or adaptation of SBIRT-And code to the Needs & Resources of Those Served by the Organization.
Evidence Strength & Quality	Definition: Patient’s and caregivers’ perceptions of the quality and validity of evidence supporting the belief that the innovation SBIRT-A will have desired outcomes. Inclusion Criteria: Include statements regarding awareness of evidence and the strength and quality of evidence. Include statements about the absence of evidence or a desire for different types of evidence, such as pilot results instead of evidence from the literature. Exclusion Criteria: Exclude or double code statements regarding the receipt of evidence as an engagement strategy for intervention among participants.
Complexity	Definition: Perceived complexity of SBIRT, reflected by duration, scope, radicalness, disruptiveness, centrality, and intricacy and number of steps required to implement. Inclusion Criteria: Code statements regarding the complexity of SBIRT-A. Include statements regarding confidentiality and maintaining patient privacy. Exclusion Criteria: Exclude statements regarding the complexity of implementation and code to the appropriate CFIR code (e.g., difficulties related to space are coded to Available Resources and difficulties related to engaging participants in a new program are coded to Engaging: hospital staff/providers).
Design Quality & Packaging	Definition: Perception of the SBIRT-A materials. Inclusion Criteria: Include statements regarding the quality of SBIRT materials. Include statements discussing accessibility of materials to use SBIRT. Exclusion Criteria: Exclude statements regarding the receipt of materials as an engagement strategy and code to Intervention Participants. Example Interview Question: What supports such as online resources, marketing materials, or a tool kit would you need to help you implement and use SBIRT-A?
Patient Needs & Resources	Definition: The extent to which the needs of those served by Lurie Children’s Hospital (e.g., patients & family), as well as barriers and facilitators to meet those needs, are accurately known and prioritized by Lurie Children’s Hospital. Inclusion Criteria: Include statements demonstrating (lack of) awareness of the needs and resources of patients at Lurie. Include statements discussing how well SBIRT-A could meet the needs of the patients and their caregivers. Analysts may be able to infer the level of awareness based on statements about: A. Perceived need for the intervention based on the needs of patients and if the intervention will meet those needs; B. Barriers and facilitators of Lurie patients to participating in the intervention; B. Participant feedback on the intervention, i.e., satisfaction and success in a program. Include statements that capture whether or not awareness of the needs and resources of patients served by Lurie influenced the implementation or adaptation of SBIRT-A. Exclusion Criteria: Exclude statements that demonstrate a strong need for SBIRT-A and/or that the current situation is untenable and code to Tension for Change. Exclude statements related to engagement strategies and outcomes, e.g., how SBIRT-A patients became engaged with the SBIRT-A, and code to Engaging: SBIRT-A Patients.
Patient Culturally Relevant Factors	Definition: Individual characteristics of patients and their caregivers that influence their interaction with the healthcare system, Lurie Children’s Hospital, and/or their provider. Inclusion Criteria: Discussion of the following: Medical mistrust (self or child/caregiver). Medical mistrust of peers—social influences from others that have medical mistrust. Health literacy of self or child/caregiver. Expectations for provider interactions. Exclusion Criteria: Do not include descriptions of patient/caregiver-clinician interaction that HAPPENED related to SBIRT and code those as clinical interaction.
Physical Structures	Definition: Physical and environmental factors that support or inhibit easy, complete, engagement with the healthcare system, Lurie Children’s Hospital or clinicians. Inclusion Criteria: Discussion of The location of the hospital. The physical building of LCH (both treatment and other spaces). Includes descriptions of both availability and state of these structures as well as how they make the person feel. Inclusive signage, forms, etc., using preferred languages. Availability of resources in the community.
Sociopolitical forces	Definition: Policies and procedures, informal or formal, in our national and local governments that systemically inhibit or promote equitable health treatment for substance use. Inclusion Criteria: Discussion of the following: Policies; political support; laws. Local cultural movements (e.g., local elections or media campaigns). Social movements (e.g., Black Lives Matter, Deplorables). Structural discrimination (e.g., racism, ableism, classism, heterosexism, transphobia). Exclusion Criteria: Code whether this is perceived by the coder or the participant being interviewed. Double code sociopolitical forces with recipient cultural factors if participant describes internalized belief that stems from structural discrimination of a particular group or stigma about SBIRT-A.

### Appendix A.2

**Table A2:** Themes and Example Concepts by CFIR and HEIF Domains.

Theme	Domains	Concepts Mentioned	
		A-CMCs	Caregivers
Sociopolitical context shaping AOD use disclosure	Sociopolitical forces—HEIF		A-CMCs glorify casual use, but there is stigma around “having a problem” or those who use/need help with drug and alcohol use.
			Prevalence and influence of social media contribute to AOD.
			Substances are readily available to A-CMCs.
	Knowledge & Belief AOD—CFIR	Influence of parents’ AOD use.	The messaging for A-CMCs can be difficult—your prescribed medications/drugs are “good” and you need to take them on time, but these other medications/drugs are “bad”.
		Some, but not all, kids use AOD.	Prevalence and availability of drugs is higher for kids in critical care (e.g., painkillers after a procedure).
	Knowledge & Belief AOD—CFIR	A-CMCs are annoyed that parents drink and smoke and do drugs but tell them not to.	Concerns that A-CMCs are not developed enough to use judgment adults would.
		Prescribed medications can be addictive.	A-CMCs need clear information about the specific risks of AOD use as they assume increasing responsibility for managing their own healthcare.
			Concerns over the lethality of AODs accessible to A-CMCs.
Structural and workflow conditions shaping inpatient SBIRT	Knowledge & Belief SBIRT—CFIR	Be clear with A-CMCs that answers about AOD are important for their treatment—not what providers think of them as “human beings”.	Contextualize the discussion with A-CMCs around their care—make sure they know the reason for providers asking about AOD use and how it relates to their health.
		It is important to ask about AOD use, but admission is overwhelming and A-CMCs might feel more inclined to limit disclosure at that time.	It should be clear that screening is necessary.
		A-CMCs may perceive AOD use screening as interrogative, increasing the likelihood of nondisclosure due to fear of getting into trouble.	Caregivers want A-CMCs to trust clinicians enough to respond honestly and to have confidence that appropriate help will follow if needed.
		SBIRT is perceived as appropriate in the inpatient hospital setting because assessing substance use is directly relevant to informing A-CMCs’ medical care.	Primary goal is optimizing A-CMCs’ care; therefore, providers should ask caregivers to leave when necessary to create a space that supports honest disclosure.
		AOD use screening is perceived as less invasive when providers frame questions in terms of potential interactions with prescribed medications.	Answering online or text-based formats may facilitate more honest disclosure by reducing the discomfort associated with face-to-face reporting.
			Prior consent should be obtained before proceeding.
			Would feel reassured and supportive of AOD screening, because someone is proactively addressing substance use.
	Patient Needs & Resources—CFIR	Important to have private time with provider	AOD can compound other preexisting vulnerabilities.
			Patients represent diverse backgrounds, contexts, and experiences, which may make it challenging to implement SBIRT in a way that is both broadly applicable and responsive to individual needs.
			The salience of AOD concerns will vary depending on the broader challenges the family is facing.
	Intervention Participants—CFIR	Asking about AOD use in the hospital, in relation to potential interactions with prescribed medications, is perceived as appropriate and clinically relevant.	Digital tools could offer clear, tangible benefits to adolescents.
		Asking too many times about AOD use can be invasive.	Suggests that a virtual format might feel more comfortable for A-CMCs.
		Unable to use technology because parent controls access to the device.	
		AOD screening should not occur during acute emergency or when the patient is fatigued. Wait to screen until medical concerns are stable.	Believes A-CMCs may initially deny AOD use but might disclose more honestly if asked once they feel settled and comfortable.
		Provider empathy and confidentiality discussions can mitigate feelings of judgment	Perceives messages delivered by peers or peer-adjacent messengers, rather than parents, as most effective.
		Hospital settings may be able to address questions about AODs in a more confidential and patient-centered manner than schools.	Notes that parental presence may limit A-CMCs’ honesty. But believe that A-CMCs are more likely to respond honestly when they feel comfortable and have been asked multiple times.
		Having a direct communication channel with the clinical team is perceived as useful.	Suggests that text-based formats may facilitate more honest responses by allowing A-CMCs to answer without direct eye contact.
		Caregivers regularly use the patient portal (MyChart).	Believe adolescents would likely complete a brief survey delivered through MyChart.
		A-CMCs typically do not use MyChart during hospitalization because they have more direct access to caregivers and clinicians.	Notes that A-CMCs are unlikely to use MyChart unless it is extremely easy to access and navigate.
Confidentiality, autonomy, and culturally grounded trust within inpatient SBIRT	Clinical Encounter—HEIF	Not fully understanding confidentiality even after it was explained by clinicians	Perceived certain physicians as uncomfortable with patient’s disability.
		Fear of shame or getting into trouble for honest disclose of AOD use	Some physicians have displayed discomfort with disability.
		Perception that physicians prioritized physical health while neglecting mental health	Noted that clinicians often lacked sufficient time to explore concerns in depth or provide thorough explanations.
		Positive feelings when physicians took time to listen to them, especially when their concerns were prioritized alongside or above those of their parents	Some providers seemed unsure how to address A-CMCs’ needs so referred them elsewhere, creating additional steps in care.
		Praised child life specialists for helping them feel comfortable and informed, and for ensuring that their voices were heard	Experiences with clinicians varied; some physicians were described as receptive and empathetic, whereas others were perceived as more business-like or transactional.
		Examples of broken trust, including not being informed about what would happen during procedures, perceived lack of honesty about lab results, clinicians sharing complete information only with parents rather than with the adolescent, and situations in which they felt the doctor did not help.	
	Complexity—CFIR	Confidentiality was often not discussed until ages 12–13 and was initially explained only briefly	Beginning around ages 12–13, caregivers are typically asked to leave the room so that A-CMCs can privately answer questions and other sensitive topics.
		Clinicians should clearly explain confidentiality and reassure A-CMCs that they will not be punished for what they disclose	Confidentiality is explained to patients during the inpatient intake process.
		Parent presence strongly influenced A-CMCs’ comfort with answering sensitive questions; however, asking A-CMCs to request that parents leave was viewed as awkward. Suggest that clinicians proactively ask caregivers to step out, rather than placing this responsibility on A-CMCs.	Around ages 12–13, caregivers are required to obtain the adolescent’s consent to access medical records through the patient portal (e.g., MyChart).
		Confidentiality was described as critically important.	
		A mixed approach that combines written (e.g., paper-based) screening with follow-up conversation with a provider was viewed as preferable.	
	Patient Culturally Relevant Factors—HEIF	Clinicians, rather than A-CMCs, asking caregivers to leave the room enhanced confidentiality and honesty while reducing concern about appearing disrespectful to caregivers.	Distinguished between trust providers vs. trust in the institutional culture, noting that clinicians generally act in patients’ best interests but operate within systems that are difficult to change.
		Speaking privately with a physician or nurse was generally perceived as positive, but A-CMCs noted ongoing discomfort because caregivers might later ask about the content of these conversations.	Desire for their A-CMC to trust providers and respond honestly to clinical questions.
		Mixed views on paper-based surveys—some perceived as more private and less intrusive, others worried that limited explanation of confidentiality could discourage honest responses. Conversation with a provider who can clarify confidentiality was viewed as preferable.	Physicians should address the patient directly to promote adolescents’ sense of being heard and to support the development of self-advocacy and confidence.
		Adolescents expected to be treated with respect by members of the care team.	The ability to select or maintain continuity with a preferred care team viewed as fostering a stronger, more positive relationship with the hospital.
		Humor and light conversation were described as helpful strategies for making serious topics feel less overwhelming or frightening.	Baseline of trust in the institution, with trust highly dependent on individual providers.
		Trust was often strongest with the usual care team; when new clinicians were involved (e.g., on weekends or during staffing changes), adolescents found it more difficult to trust them in the absence of an established rapport.	Receptiveness, understanding, empathy, and open-mindedness were identified as key behaviors that enhance trust, whereas experiences of misinformation were seen as undermining it.
			Negative experiences are shared within networks, which may influence future decisions about care at the institution.
			Want clinicians to listen to and understand what occurs in their lives outside of the clinical encounter.
			Failures to obtain an accurate or timely diagnosis were described as eroding trust in providers and the institution.
Structural barriers and enablers shaping SBIRT follow-up	Physical Structures HEIF	Simply being in the hospital can feel emotionally overwhelming due to prior experiences in that setting.	Financial burden for families of A-CMCs is substantial. The cost of inpatient stays and parking are compounded by the high frequency of hospital visits.
		Noted the presence of signage promoting diversity, equity, inclusion, accessibility, social justice, and health equity within the hospital.	Physical environment and clinical workflows often do not adequately account for the needs of children with disabilities.
		Attending appointments was described as time-intensive, with travel and visit logistics often consuming most of the day.	Limited resources for follow-up, including prolonged wait times for psychiatric services.
			Hospital location difficult to access, requiring lengthy trips via highway or public transportation and, when appointments could not be consolidated, multiple separate visits.
			Parking is limited and expensive.
			Satellite clinics were also viewed as inaccessible, as they are primarily concentrated in downtown and northern areas.

## References

[1] Roberts R.E., Roberts C.R., Xing Y. (2007). Comorbidity of substance use disorders and other psychiatric disorders among adolescents: Evidence from an epidemiologic survey. Drug Alcohol Depend..

[2] Gray K.M., Squeglia L.M. (2018). Research Review: What have we learned about adolescent substance use?. J. Child Psychol. Psychiatry.

[3] Grant J.D., Scherrer J.F., Lynskey M.T., Agrawal A., Duncan A.E., Haber J.R., Heath A.C., Bucholz K.K. (2012). Associations of alcohol, nicotine, cannabis, and drug use/dependence with educational attainment: Evidence from cotwin-control analyses. Alcohol. Clin. Exp. Res..

[4] Heradstveit O., Skogen J.C., Hetland J., Hysing M. (2017). Alcohol and Illicit Drug Use Are Important Factors for School-Related Problems among Adolescents. Front. Psychol..

[5] Substance Abuse and Mental Health Services Administration (2014). Alcohol and Drug Combinations Are More Likely to Have a Serious Outcome Than Alcohol Alone in Emergency Department Visits Involving Underage Drinking, in The DAWN Report.

[6] Suris J.C., Michaud P.-A., Akre C., Sawyer S.M. (2008). Health risk behaviors in adolescents with chronic conditions. Pediatrics.

[7] Wisk L.E., Weitzman E.R. (2016). Substance Use Patterns Through Early Adulthood: Results for Youth with and Without Chronic Conditions. Am. J. Prev. Med..

[8] Weitzman E.R., Ziemnik R.E., Huang Q., Levy S. (2015). Alcohol and Marijuana Use and Treatment Nonadherence Among Medically Vulnerable Youth. Pediatrics.

[9] Weitzman E.R., Salimian P.K., Rabinow L., Levy S. (2019). Perspectives on substance use among youth with chronic medical conditions and implications for clinical guidance and prevention: A qualitative study. PLoS ONE.

[10] Balfe M. (2009). Healthcare routines of university students with Type 1 diabetes. J. Adv. Nurs..

[11] Kossowsky J., Magane K.M., Levy S., Weitzman E.R. (2021). Marijuana Use to Address Symptoms and Side Effects by Youth with Chronic Medical Conditions. Pediatrics.

[12] Breslow R.A., Dong C., White A. (2015). Prevalence of alcohol-interactive prescription medication use among current drinkers: United States 1999 to 2010. Alcohol. Clin. Exp. Res..

[13] Weitzman E.R., Magane K.M., Wisk L.E., Allario J., Harstad E., Levy S. (2018). Alcohol Use and Alcohol-Interactive Medications Among Medically Vulnerable Youth. Pediatrics.

[14] Goossens E., Bovijn L., Gewillig M., Budts W., Moons P. (2016). Predictors of Care Gaps in Adolescents with Complex Chronic Condition Transitioning to Adulthood. Pediatrics.

[15] Wisk L.E., Finkelstein J.A., Sawicki G.S., Lakoma M., Toomey S.L., Schuster M.A., Galbraith A.A. (2015). Predictors of timing of transfer from pediatric- to adult-focused primary care. JAMA Pediatr..

[16] Jensen P.T., Paul G.V., LaCount S., Peng J., Spencer C.H., Higgins G.C., Boyle B., Kamboj M., Smallwood C., Ardoin S.P. (2017). Assessment of transition readiness in adolescents and young adults with chronic health conditions. Pediatr. Rheumatol..

[17] Substance Abuse and Mental Health Services Administration (2013). Systems-Level Implementation of Screening, Brief Intervention, and Referral to Treatment, in Technical Assistance Publication (TAP) Series.

[18] Mitchell S.G., Gryczynski J., O’GRady K.E., Schwartz R.P. (2013). SBIRT for adolescent drug and alcohol use: Current status and future directions. J. Subst. Abus. Treat..

[19] Harris S.K., Herr-Zaya K., Weinstein Z., Whelton K., Perfas F., Castro-Donlan C., Straus J., Schoneman K., Botticelli M., Levy S. (2012). Results of a statewide survey of adolescent substance use screening rates and practices in primary care. Subst. Abus..

[20] Williams F.S., Welch S., Gopagani N., Zylka I., Curtis K., Franklin P., Becker S. (2026). Health professional perspectives on integrating substance use services into pediatric hospitals for adolescents with chronic medical conditions. Discov. Health Syst..

[21] O’Mahony L., O’mAhony D.S., Simon T.D., Neff J., Klein E.J., Quan L. (2013). Medical complexity and pediatric emergency department and inpatient utilization. Pediatrics.

[22] Mah S.S., Teare G.F., Law J., Adhikari K. (2024). Facilitators and barriers for implementing screening brief intervention and referral for health promotion in a rural hospital in Alberta: Using consolidated framework for implementation research. BMC Health Serv. Res..

[23] Sterling S., Kline-Simon A.H., Wibbelsman C., Wong A., Weisner C. (2012). Screening for adolescent alcohol and drug use in pediatric health-care settings: Predictors and implications for practice and policy. Addict. Sci. Clin. Pract..

[24] Hammond C.J., Parhami I., Young A.S., Matson P.A., Alinsky R.H., Adger H., Levy S., Horner M. (2021). Provider and Practice Characteristics and Perceived Barriers Associated with Different Levels of Adolescent SBIRT Implementation Among a National Sample of US Pediatricians. Clin. Pediatr..

[25] Keen A., Thoele K., Oruche U., Newhouse R. (2021). Perceptions of the barriers, facilitators, outcomes, and helpfulness of strategies to implement screening, brief intervention, and referral to treatment in acute care. Implement. Sci..

[26] Beresford B.A., Sloper P. (2003). Chronically ill adolescents’ experiences of communicating with doctors: A qualitative study. J. Adolesc. Health.

[27] Britto M.T., DeVellis R.F., Hornung R.W., DeFriese G.H., Atherton H.D., Slap G.B. (2004). Health care preferences and priorities of adolescents with chronic illnesses. Pediatrics.

[28] Lunstead J., Weitzman E.R., Harstad E., Dedeoglu F., Gaffin J.M., Garvey K.C., MacGinnitie A., Rufo P.A., Fishman L.N., Wisk L.E. (2019). Screening and Counseling for Alcohol Use in Adolescents with Chronic Medical Conditions in the Ambulatory Setting. J. Adolesc. Health.

[29] Shelton R.C., Adsul P., Oh A. (2021). Recommendations for Addressing Structural Racism in Implementation Science: A Call to the Field. Ethn. Dis..

[30] Allen M., Wilhelm A., Ortega L.E., Pergament S., Bates N., Cunningham B. (2021). Applying a Race(ism)-Conscious Adaptation of the CFIR Framework to Understand Implementation of a School-Based Equity-Oriented Intervention. Ethn. Dis..

[31] Kirk M.A., Kelley C., Yankey N., Birken S.A., Abadie B., Damschroder L. (2016). A systematic review of the use of the Consolidated Framework for Implementation Research. Implement. Sci..

[32] Damschroder L.J., Aron D.C., Keith R.E., Kirsh S.R., Alexander J.A., Lowery J.C. (2009). Fostering implementation of health services research findings into practice: A consolidated framework for advancing implementation science. Implement. Sci..

[33] Woodward E.N., Matthieu M.M., Uchendu U.S., Rogal S., Kirchner J.E. (2019). The health equity implementation framework: Proposal and preliminary study of hepatitis C virus treatment. Implement. Sci..

[34] Woodward E.N., Singh R.S., Ndebele-Ngwenya P., Castillo A.M., Dickson K.S., Kirchner J.E. (2021). A more practical guide to incorporating health equity domains in implementation determinant frameworks. Implement. Sci. Commun..

[35] O’Brien B.C., Harris I.B., Beckman T.J., Reed D.A., Cook D.A. (2014). Standards for reporting qualitative research: A synthesis of recommendations. Acad. Med..

[36] Malterud K., Siersma V.D., Guassora A.D. (2016). Sample Size in Qualitative Interview Studies: Guided by Information Power. Qual. Health Res..

[37] (2025). Dedoose.

[38] Bray E.A., Everett B., George A., Salamonson Y., Ramjan L.M. (2022). Co-designed healthcare transition interventions for adolescents and young adults with chronic conditions: A scoping review. Disabil. Rehabil..

[39] Zolfaghari E., Armaghanian N., Waller D., Medlow S., Hobbs A., Perry L., Nguyen K., Steinbeck K. (2022). Implementation science in adolescent healthcare research: An integrative review. BMC Health Serv. Res..

[40] Valencia L.S., Cromer B.A. (2000). Sexual activity and other high-risk behaviors in adolescents with chronic illness: A review. J. Pediatr. Adolesc. Gynecol..

[41] Berridge B.J., McCann T.V., Cheetham A., Lubman D.I. (2018). Perceived Barriers and Enablers of Help-Seeking for Substance Use Problems During Adolescence. Health Promot. Pract..

[42] Fisher S., Benner K., Huang H., Day E. (2024). Substance Use Screening, Brief Intervention, and Referral to Treatment in Urban Settings: Barriers and Facilitators to Implementation with Minoritized Youth. J. Sch. Health.

[43] Shaligram D., Arshad S.H., Rogers K., Caraballo A.A., Tumuluru R.V. (2024). Creating an Equitable System of Care for Minoritized Youth and Addressing Systemic and Structural Barriers. Child Adolesc. Psychiatr. Clin. N. Am..

[44] Eisenberg M.E., Toumbourou J.W., Catalano R.F., Hemphill S.A. (2014). Social norms in the development of adolescent substance use: A longitudinal analysis of the International Youth Development Study. J. Youth Adolesc..

[45] Scull T.M., Kupersmidt J.B., Parker A.E., Elmore K.C., Benson J.W. (2010). Adolescents’ media-related cognitions and substance use in the context of parental and peer influences. J. Youth Adolesc..

[46] Clement A., Corcoran E., Jackson K.M., Gabrielli J. (2025). “If You Need to Light Up … You Gotta Do What You Gotta Do”: A Qualitative Study of Adolescent Attitudes Towards Cannabis Use and Comparison with Alcohol Attitudes. Cannabis.

[47] Warren J.C., Smalley K.B., Barefoot K.N. (2015). Perceived ease of access to alcohol, tobacco and other substances in rural and urban US students. Rural. Remote Health.

[48] Gryczynski J., Mitchell S.G., Schwartz R.P., Kelly S.M., Dušek K., Monico L., O’GRady K.E., Brown B.S., Oros M., Hosler C. (2019). Disclosure of Adolescent Substance Use in Primary Care: Comparison of Routine Clinical Screening and Anonymous Research Interviews. J. Adolesc. Health.

[49] Wombacher K., Darnell W.H., Harrington N.G., Scott A.M., Martin C.A. (2022). Identifying Conversational Strategies for Psychiatrists in Discussing Substance Use with Adolescent Patients. Health Commun..

[50] Lothen-Kline C., Howard D., Hamburger E., Worrell K., Boekeloo B. (2003). Truth and consequences: Ethics, confidentiality, and disclosure in adolescent longitudinal prevention research. J. Adolesc. Health.

[51] Thrall J.S., McCloskey L., Ettner S.L., Rothman E., Tighe J.E., Emans S.J. (2000). Confidentiality and adolescents’ use of providers for health information and for pelvic examinations. Arch. Pediatr. Adolesc. Med..

[52] Weddle M., Kokotailo P.K. (2005). Confidentiality and consent in adolescent substance abuse: An update. AMA J. Ethics.

[53] Levy S.J., Kokotailo P.K., Committee on Substance Abuse (2011). Substance use screening, brief intervention, and referral to treatment for pediatricians. Pediatrics.

[54] Hardin H.K., Bender A.E., Hermann C.P., Speck B.J. (2021). An integrative review of adolescent trust in the healthcare provider relationship. J. Adv. Nurs..

[55] Theis R.P., Malik A.M., Thompson L.A., Shenkman E.A., Pbert L., Salloum R.G. (2019). Considerations of Privacy and Confidentiality in Developing a Clinical Support Tool for Adolescent Tobacco Prevention: Qualitative Study. JMIR Form. Res..

[56] Ozechowski T.J., Becker S.J., Hogue A. (2016). SBIRT-A: Adapting SBIRT to Maximize Developmental Fit for Adolescents in Primary Care. J. Subst. Abus. Treat..

[57] Malloy J., Partridge S.R., Kemper J.A., Braakhuis A., Roy R. (2023). Co-design of digital health interventions with young people: A scoping review. Digit. Health.

[58] Bevan Jones R., Stallard P., Agha S.S., Rice S., Werner-Seidler A., Stasiak K., Kahn J., Simpson S.A., Alvarez-Jimenez M., Rice F. (2020). Practitioner review: Co-design of digital mental health technologies with children and young people. J. Child Psychol. Psychiatry.

[59] Díaz Hurtado R., Goti J., Magallón-Neri E., Mateus-Gómez S., Ilzarbe D., Castro-Fornieles J. (2024). Integrated vs. parallel treatment in adolescents with substance use and comorbid disorders: A randomized trial. Adicciones.

